# Lesion Size Is Exacerbated in Hypoxic Rats Whereas Hypoxia-Inducible Factor-1 Alpha and Vascular Endothelial Growth Factor Increase in Injured Normoxic Rats: A Prospective Cohort Study of Secondary Hypoxia in Focal Traumatic Brain Injury

**DOI:** 10.3389/fneur.2016.00023

**Published:** 2016-03-07

**Authors:** Eric Peter Thelin, Arvid Frostell, Jan Mulder, Nicholas Mitsios, Peter Damberg, Sahar Nikkhou Aski, Mårten Risling, Mikael Svensson, Maria Cristina Morganti-Kossmann, Bo-Michael Bellander

**Affiliations:** ^1^Department of Clinical Neuroscience, Karolinska Institutet, Stockholm, Sweden; ^2^Science for Life Laboratory, Department of Neuroscience, Karolinska Institutet, Stockholm, Sweden; ^3^Karolinska Experimental Research and Imaging Center, Karolinska Universitetssjukhuset Solna, Stockholm, Sweden; ^4^Department of Neuroscience, Karolinska Institutet, Stockholm, Sweden; ^5^Department of Neurosurgery, Karolinska University Hospital, Stockholm, Sweden; ^6^Department of Epidemiology and Preventive Medicine, Monash University, Melbourne, VIC, Australia; ^7^Department of Child Health, Barrow Neurological Institute, Phoenix Children’s Hospital, University of Arizona College of Medicine Phoenix, Phoenix, AZ, USA

**Keywords:** traumatic brain injury, hypoxia, hypoxia-inducible factor 1, vascular endothelial growth factor A, S100B, edema, neuronal death, secondary insults

## Abstract

**Background:**

Hypoxia following traumatic brain injury (TBI) is a severe insult shown to exacerbate the pathophysiology, resulting in worse outcome. The aim of this study was to investigate the effects of a hypoxic insult in a focal TBI model by monitoring brain edema, lesion volume, serum biomarker levels, immune cell infiltration, as well as the expression of hypoxia-inducible factor-1 alpha (HIF-1α) and vascular endothelial growth factor (VEGF).

**Materials and methods:**

Female Sprague-Dawley rats (*n* = 73, including sham and naive) were used. The rats were intubated and mechanically ventilated. A controlled cortical impact device created a 3-mm deep lesion in the right parietal hemisphere. Post-injury, rats inhaled either normoxic (22% O_2_) or hypoxic (11% O_2_) mixtures for 30 min. The rats were sacrificed at 1, 3, 7, 14, and 28 days post-injury. Serum was collected for S100B measurements using ELISA. *Ex vivo* magnetic resonance imaging (MRI) was performed to determine lesion size and edema volume. Immunofluorescence was employed to analyze neuronal death, changes in cerebral macrophage- and neutrophil infiltration, microglia proliferation, apoptosis, complement activation (C5b9), IgG extravasation, HIF-1α, and VEGF.

**Results:**

The hypoxic group had significantly increased blood levels of lactate and decreased pO_2_ (*p* < 0.0001). On MRI post-traumatic hypoxia resulted in larger lesion areas (*p* = 0.0173), and NeuN staining revealed greater neuronal loss (*p* = 0.0253). HIF-1α and VEGF expression was significantly increased in normoxic but not in hypoxic animals (*p* < 0.05). A trend was seen for serum levels of S100B to be higher in the hypoxic group at 1 day after trauma (*p* = 0.0868). No differences were observed between the groups in cytotoxic and vascular edema, IgG extravasation, neutrophils and macrophage aggregation, microglia proliferation, or C5b-9 expression.

**Conclusion:**

Hypoxia following focal TBI exacerbated the lesion size and neuronal loss. Moreover, there was a tendency to higher levels of S100B in the hypoxic group early after injury, indicating a potential validity as a biomarker of injury severity. In the normoxic group, the expression of HIF-1α and VEGF was found elevated, possibly indicative of neuro-protective responses occurring in this less severely injured group. Further studies are warranted to better define the pathophysiology of post-TBI hypoxia.

## Introduction

Traumatic brain injury (TBI) is the leading cause of death and disability among young adults worldwide and is constantly increasing among the elderly (1–3). TBI consists of two processes: the initial traumatic impact at the accident scene causing primary damage to the cerebral parenchyma and blood vessels, followed by the onset of detrimental secondary insults (4), subsequently resulting in progressive cerebral deterioration (5). These insults include intracranial hypertension, hypoxia, hypotension, metabolic dysfunction, and seizures and may lead to irreversible delayed injuries that impair functional recovery after TBI (5, 6). Unconscious patients suffering from TBI are treated at specialized neuro-intensive care units where the goal is to detect and treat secondary insults to optimize cerebral recovery. Neuroradiology is used to detect intracranial pathology, magnetic resonance imaging (MRI) being superior to computerized tomography (CT) in revealing edema and ischemia after TBI (7).

A decreased level of oxygen saturation in the brain, defined as cerebral hypoxia, may be the result of obstructed airways, respiratory failure or any other injuries to lungs, chest, or associated vessels. Epidemiological studies have shown that 20–45% of TBI patients suffer from pre-hospital hypoxia (6, 8), a condition that has been correlated to worse outcome (6, 9–11). Reduced cerebral oxygenation may contribute to ischemic damage, an irreversible secondary brain injury that is frequently seen in autopsy materials of TBI patients (12). In experimental TBI, the addition of hypoxia causes an exacerbated cerebral inflammatory response (13, 14), aggravated neuronal death, and larger lesion size (14–17), having a profound effect on blood–brain barrier (BBB) dysfunction, edema formation (13, 18, 19), and increased intracranial pressure (20), as well as worse functional outcomes (13, 14, 19, 21–23). The pathophysiological impact of hypoxic insult after TBI is not fully understood. Post-traumatic hypoxia is thought to trigger a cascade of events, leading to apoptosis and tissue loss (24). A transient hypoxic state will aggravate the physiological disarray in the perilesional area, challenging the metabolic demand and energy supply for cells already in a critical state.

The inflammatory response following TBI has been shown to possess both detrimental and beneficial properties (25). This involves the activation of the innate immune system, leading to cerebral accumulation of peripheral macrophages and leukocytes as well as the mobilization of local microglia around the lesion occurring mainly during the first week following TBI (26). The complement system, a key element of the innate immune system, regulating the chemotaxis and lysis of cells, has also been shown to increase in activity specifically in brain regions adjacent to traumatic contusions in both rats (27) and humans (28).

Hypoxia-inducible factor-1 alpha (HIF-1α), a transcription factor involved in oxygen homeostasis (29), provides an adaptive response to the pathological conditions occurring during hypoxia (30). When oxygen concentration drops, HIF-1α triggers the upregulation of several genes, which may result in either detrimental cellular pathways such as apoptosis (31), or neuro-protective effects including angiogenesis through the upregulation of vascular endothelial growth factor (VEGF) (32), erythropoiesis *via* the induction of erythropoietin (33, 34) as well as mitochondrial integrity (35) and cell survival (36).

S100B, a protein present primarily in perivascular astrocytes (37), is measured in blood early after TBI and used as a reliable biomarker to detect and grade the severity (38) of brain damage in clinical practice (39). High levels of S100B have been correlated to an unfavorable outcome (40) while its delayed elevation has been associated primarily with the development of secondary ischemia (41, 42).

Although numerous studies have addressed the pathological consequences of post-traumatic hypoxia in models of TBI (18, 19, 21, 43–45), several aspects aggravated by this insult remain obscure. The main focus of hypoxic TBI models has been primarily to assess changes in neuronal loss (22, 46), with only a few studies analyzing the underlying pathophysiological mechanisms (13, 14). The neuro-inflammatory response has been studied in diffuse hypoxic TBI (14), but to the best of our knowledge not in a focal hypoxic TBI model. In addition, complement activation, S100B monitoring, and HIF-1α and VEGF expression have never before been examined in hypoxic-TBI. Moreover, while the MRI technique has been utilized acutely after TBI (19, 47), extensive follow-up 4 weeks has never been performed.

By using an animal model of focal (controlled cortical impact, CCI) hypoxic TBI, we expect to better elucidate the burden of the secondary hypoxic insult by providing in-depth changes in radiological, histological, metabolic, inflammatory, and serum markers of injury.

### Aims

The primary aim of our study was to employ a hypoxic focal TBI rat model using the CCI paradigm and to explore the effect of post-traumatic hypoxia on brain morphological changes including lesion progression and vascular/cytotoxic edema, serum profiles of the biomarker S100B, alteration of metabolic parameters (lactate), brain histopathological features such as neuronal survival, leukocyte and macrophage infiltration, all of which were compared to normoxic rats, over 4 weeks.

## Materials and Methods

### Animals

All procedures were conducted following approval by the ethical committee of the Swedish Board of Agriculture (applications #N369/12 and #N126/13). Seventy-three female Sprague-Dawley rats were included. On the day of surgery rats weighed an average 251 g (~15 weeks of age). Animal were housed in a 12-h light/dark cycle with food and water *ad libitum* and kept throughout the study at room temperature (21 ± 1°C) with normal air humidity.

### Anesthesia and Oxygen Monitoring – Set Up

The animals were anesthetized using a mixture of 5% isoflurane in 22% O_2_/78% N_2_, then intubated using the plastic tubing of a 16 gage angiocatheter (KD Medical, Berlin, Germany), and mechanically ventilated (Rodent ventilator, Ugo Basile, Gemonio, Italy) with a maintenance dose of 2–3% isoflurane in 22% O_2_/78% N_2_. A re-built pediatric gas mixer with vaporizer was used (Dameca A/S, currently Philips Electronics, Amsterdam, Netherlands and Penlon, Oxford, UK). A pulsoximetry device was attached to the rat’s right back paw to monitor heart rate and oxygen saturation (MouseSTAT™, Kent Scientific, Torrington, CT, USA). In order to certify that a correct amount of oxygen was inhaled, a portable oxygen analyzer was used (TED 60-T, Teledyne Electronic Devices, Thousand Oaks, CA, USA). The respiratory rate was kept 90 bpm and the tidal volume to 2 mL, according to normal levels in an anesthetized spontaneously breathing rat (48). The anesthesiology set-up is illustrated in Figure S1 in Supplementary Material.

### Surgery – Procedure

Following adequate sedation, local anesthesia [0.15 mL of bupivacaine (Marcaine^®^) 0.25%] was injected subcutaneously in the scalp above the cranial midline, while buprenorphine (Temgesic^®^) (0.05 mg/kg) and carprofen (5 mg/kg) were injected subcutaneously in the abdominal region, in order to provide analgesia. Eye-gel [containing fusidic acid (Fucithalmic^®^)] was applied to protect the eyes, and isotonic saline (NaCl 9 mg/mL) was used to rinse and clean the scalp wound throughout the experiment. Following pre-medications, the rat was placed on a heating pad (Temperature Control Unit HB 101/2, Panlab, Harvard Apparatus, Barcelona, Spain) attached to a stereotaxic frame (Model 900, Agnthos, Stockholm, Sweden). During the surgical procedure, the rats’ body temperature was maintained within a normal range (average 37.2°C) (49).

**Figure 1:**
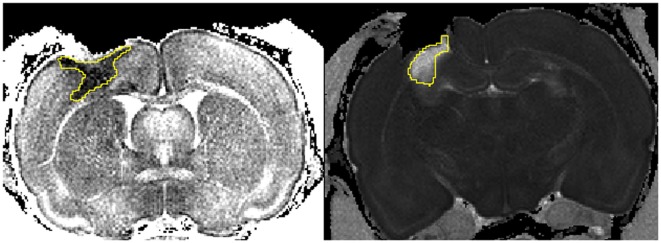
**Cytotoxic and vasogenic edema following injury**. Left picture depicts the area of decreased apparent diffusion coefficient (ADC) indicating cytotoxic edema (1 day hypoxic). Right picture shows the area affected by an increase in ADC highlighting vasogenic edema (14 days normoxic). The freehand tool in ImageJ was used to define the areas (yellow).

Using a surgical drill with a diamond tip of 0.5 mm diameter (Microspeed 317 IN; Silfradent, Forli, Italy), a portion of the parietal bone over the right hemisphere was removed, at 3.5 mm right of the central suture and 4.5 mm posterior to lambda. For precision, the procedure was performed under a surgical microscope (Wild Heerbrugg M3C Stereozoom Microscope, Leica, Wetzlar, Germany). A piston of 3 mm in diameter was used to impact a 3-mm deep lesion in the right parietal lobe, producing an injury previously defined as a “severe TBI” (50) (Figure 1). A commercially available CCI device was employed (TBI 0310, Precision Systems and Instrumentation LLC, Lexington, KY, USA), as described in previous studies (51, 52). The lesion produced mimics the conditions seen in the clinical setting of a focal TBI (53). The wound was sutured using Vicryl 4-0 (Ethicon, Johnson & Johnson, New Brunswick, NJ, USA).

Following trauma, the animals were ventilated with either a hypoxic (11% O_2_/88% N_2_) (AGA AB, Linde, Munich, Germany) or normoxic (22% O_2_/78% N_2_) gas mixture for 30 min, with an appropriate amount of isoflurane to maintain sedation (1–1.5%). This protocol has previously been shown to induce a systemic hypoxia, resulting in an oxygen saturation of 47 ± 4.3% and pO_2_ of 48.5 ± 3.8 mmHg (6.47 ± 0.5 kPa), and a brief hypotensive episode, with mean arterial pressure (MAP) dropping to 69.5 ± 29.5 mmHg after 15 min (21, 22). A previous study, by a member of our group, has determined that partial oxygen pressure, oxygen saturation, and blood pressure normalize 15 min after the completion of the hypoxic phase (14). Our set-up was similar to a hypoxic TBI model of diffuse injury used by the collaborating group (13), though now applied to a CCI model generating a focal, severe injury.

Following 30 min of hypoxia/normoxia, the animals were placed on a heat pad until fully awake (~30 min) before returning to their home cages. The mortality rate for the experiment was about 20% during trauma/hypoxia induction, and no deaths occurred post-surgery.

In sham animals, all procedures including craniotomy, apart from the impact, were conducted. This included a position calibration performed before every impact by the impactor, applying ~0.6 g of weight to the dura before the impact (as default by the manufacturer).

A video demonstrating the surgical and post-operative procedure is provided in Video S1 in Supplementary Material.

### Preoperative Monitoring

The MouseSTAT device (Kent Scientific, Torrington, CT, USA) attached to the rat’s back right paw was used to monitor physiological parameters throughout the experimental procedure, including pulse, saturation, and perfusion index. According to the manufacturer, perfusion index is a value indicating the strength of the plethysmographic signal at the sensor site. The greater the number, the greater the level of blood flow is at the sensor site. Data were recorded for the whole experimental procedure as well as during the 30 min of normoxic or hypoxic ventilation.

### Arterial Blood Sampling

Following the normoxic/hypoxic period, a small incision was performed along the midline of the tail base to expose the artery. A heparin-coated blood gas syringe was inserted, and about 2 mL of blood was drawn and immediately used for blood gas analysis (ABL800 FLEX analyzer, Radiometer Medical, Brønshøj, Denmark). The wound was readily stitched using Vicryl 4-0.

### Euthanasia and Experimental Groups

Traumatic brain injury rats were divided into two groups: a group (*n* = 25) subjected to a normoxic gas mixture and a second group (*n* = 25) exposed to the hypoxic gas mixture. Rats of both groups were euthanized at 1, 3, 7, 14, and 28 days following injury (*n* = 5 per day) using an 80 mg/kg intraperitoneal pentobarbital injection. For control, we included normoxic (*n* = 10) and hypoxic (*n* = 10) sham rats (day 1 and 7 post-injury, *n* = 5 per group) and naive rats (*n* = 3), resulting in a total of *n* = 73 rats.

### Blood Collection and Isolation of Serum

After the animal received a lethal dose of pentobarbital, the heart was punctured using an angiocatheter (18G, KD Medical, Berlin, Germany) and blood was collected in two test tubes (2 × 1.5 mL test tube, 3810X, Eppendorf, Hamburg, Germany). The tubes were placed vertically for about 1 h to allow fractionation. Afterward, the blood was centrifuged (Spectrafuge16M^®^, Labnet International, Edison, NJ, USA) for 10 min at 10,000 × *g* [about 11,000 rotations per minute (RPM)], the serum was collected, and the frozen in −80°C.

### S100B Measurement and Analysis

A commercially available S100B ELISA kit was used (CanAg EIA S100, Fujirebio Diagnostics AB, Göteborg, Sweden). Serum samples where thawed overnight at 4°C and were transferred (50 μL per well) to a pre-coated 96-well plate (in duplicates) and run according to manufacturer’s protocol. The results were analyzed using a Multiskan EX and Ascent Software V2.6 (ThermoLabSystems, Thermo Scientific, Waltham, MA, USA). Absorbance was measured at 620 nm, and cubic spline curve fit method was used to fit the absorbance as recommended by the manufacturer. Extrapolation was used for sample levels outside the calibration reference levels. Outliers (deviating more than 5 SD) were excluded (*n* = 2) (both normoxic, day 7), as well as animals with an inadequate sample volume (*n* = 1) (hypoxic, day 7).

### *Ex Vivo* Magnetic Resonance Imaging

Following euthanasia, *ex vivo* MRI scanning was performed on two animals from each time point per group (normoxia and hypoxia on days 1, 3, 7, 14, 28, and sham on day 1). A total of 23 rats were scanned (one hypoxic brain on day 28 could not be used due to procedural errors). Each brain was fixed in formalin (as described in Section “Brain Preparation for Immunofluorescence”) and positioned in a syringe filled with Fomblin (Solvay Solexis, Brussels, Belgium) to avoid image artifacts due to susceptibility mismatch while providing a dark background. A horizontal 9.4-T magnetic resonance scanner (Agilent, Yarnton, UK) equipped with a millipede coil of 30-mm inner diameter coil was used for data acquisition. The brains were scanned overnight using a diffusion-weighted spin echo with gradients applied in 30 different directions as well as a reference image with the diffusion encoding gradients set to 0 (tr = 2.5 s, te = 20.83 ms, nex = 2, matrix = 192 × 192, FOV = 19.2 mm × 19.2 mm, 51 continuous 0.5 mm thick slices). The data were zero-filled to 256 × 256 points before Fourier transformation. The diffusion-weighted data were interpreted using the diffusion tensor model. The T2-weighted reference image, the fractional anisotropy map, and the diffusion-weighted image were analyzed further as described below.

### MRI – Lesion Area Measurement

The software ImageJ^®^ (1.48v, NIH, Bethesda, MD, USA) was used to map the lesion area using the “Polygon selections.” T2 images were taken at 7, 14, and 28 days post-TBI since they had well-defined lesion areas. All sections with a lesion were assessed and the largest lesion area per brain was chosen for quantification.

### MRI – Edema Quantification

The freehand tool in ImageJ was used to detect the pericontusional area with an increased apparent diffusion coefficient (ADC) signaling indicating vasogenic edema and a decreased ADC indicating cytotoxic edema (54–56) (Figure 1). All MRI sections including edema were quantified and displayed as max value (square millimeter) per animal. The lesion cavity was not included in the edema areas.

### Brain Preparation for Immunofluorescence

After euthanasia, transcardial perfusion was performed using 300 mL NaCl (9 mg/mL) (37°C) followed by a perfusion-fixation over 10 min with 300 mL chilled paraformaldehyde (PFA) (4°C) (P6148, 4%, Sigma-Aldrich, Germany). The rat was finally decapitated and the brain immerged in a 4% formaldehyde solution for 1 h. Following a rinse step, the brain was stored in a PBS Mill solution (in house) containing 0.1% NaN_3_ (sodium azide). Prior to sectioning, the brain was immerged in 30% sucrose solution for 3 days and then immediately frozen to −80°C. Using a cryostat (Cryostar NX70, ThermoScientific, Waltham, MA, USA) brains were cut in coronal sections of 14 μm thickness and placed on Superfrost^®^ glass slides. The sections included the whole lesion (*n* = 100 slides per brain with two to three sections per slide). Subsequently, the tissue was air-dried and then frozen at −20°C.

### Immunofluorescence – Antibodies

Commercially available primary and secondary antibodies were used as presented in Table 1. To best visualize the immune cell activation to cerebral injury, we chose the following antibodies: CD68/ED1 for macrophages (and activated microglia) (14, 57); CD34 for proliferating microglia (58) and CD43/W3/13 for neutrophils; Activated caspase 3 antibody for cell apoptosis (59); and NeuN (Neuronal Nuclei/Fox3) for neurons. In addition, we used a specific antibody against the membrane attack complex (C5b-9) for detection of end stage activation of the complement cascade (28), anti-HIF-1α antibody to monitor changes in this transcription factor (60) as well as an antibody against VEGF alpha to detect increased angiogenesis (61) and anti-rat IgG to quantify the extent of BBB disruption (18). DAPI (4′,6-diamidino-2-phenylindole) was chosen as cellular counterstain. For control, all secondary antibodies were tested with omission of the primary antibodies resulting in no significant background noise, as seen in Figures S2A–D in Supplementary Material.

**Table 1:** **Primary and secondary antibodies**.

Antibody	Manufacturer	Product-ID	Dilution	Secondary	Exposure time	Cut-off
DAPI	Life Technologies	P36931	NA	(Included in mounting medium)	0.08	NA
NeuN	Millipore	ABN90	1:8000	Cy3, anti-guinea pig, 706-165-148, Jackson Laboratories, 1:200	0.12	NA
ED1 (CD68)	Serotec	MCA341R	1:2000	Cy5, anti-mouse, 715-175-150, Jackson Laboratories, 1:200	0.24	10
Activated Caspase 3	Abcam	AB2302	1:300	Cy2, anti-rabbit, 711-545-152, Jackson Laboratories, 1:200	0.24	22
C5b-9	Dako	M0777	1:500	Cy5 (anti-mouse)^a^	0.08	100
Leukosialin (CD43, W3/13)	Serotec	MCA54	1:250	Cy5, anti-mouse, 715-175-150, Jackson Laboratories, 1:200	0.28	36
VEGF-Alpha	Antibodies online	AA-27190/ABIN1078647	1:250	Cy5, anti-mouse, 715-175-150, Jackson Laboratories, 1:200	0.28	24
IgG	Antibodies online	ABIN458741	1:500	Cy3.5, anti-goat, 705-585-147, Jackson Laboratories, 1:200	0.20	90
HIF-1α	Millipore	MAB5382	1:250	Cy5, anti-mouse, 715-175-150, Jackson Laboratories, 1:200	0.22	31
CD34	Antibodies online	ABIN1719820	1:250	Cy2, anti-rabbit, 711-545-152, Jackson Laboratories, 1:200	0.32	90

*Primary and secondary antibodies used in immunofluorescence labeling. Exposure time indicates the “integration time” for the microscope to scan each section of the slide. “Cut-off” is the threshold used in ImageJ, antibody expression above this level was quantified. For C5b-9, tyramide signal amplification was used (see Tyramide Signal Amplification for Detection of Complement Factor C5b-9)*.

*^a^Was used together with tyramide signal amplification*.

### Immunofluorescence – Staining Protocol

When preparing for immunofluorescence, all slides were air-dried at room temperature and then rehydrated in phosphate buffered saline (PBS) (P4417 Sigma-Aldrich, St. Louis, MO, USA) for 15 min. Primary antibodies were diluted in 0.3% Triton X-100 (X100, Sigma-Aldrich, St. Louis, MO, USA) and 0.1% NaN_3_ in PBS pH7.4 for 16 h (4°C). Afterwards, sections were washed three times in Tris Buffered Saline solution, with Tween^®^ 20 (0.05%) (TBS-Tween 20, T9039 Sigma-Aldrich, St. Louis, MO, USA) for 15 min. The sections were blocked with a Tris–NaCl-blocking buffer (TNB-buffer, FP1020, PerkinElmer, Waltham, MA, USA) for 30 min at room temperature. This was followed by the addition of the secondary fluorescent labeled antibody mix diluted in TNB-buffer and sections incubated for 90 min at room temperature. Subsequently, the sections were washed three times in 0.05% TBS-Tween 20 for 15 min, in the dark. The sections then were immersed in 70% EtOH for 5 min before being transferred to Sudan Black (1% solution, 70%EtOH) for 10 min and then rinsed in 70% EtOH for about a minute before being mounted, using PVA/DABCO (ProLong^®^ Gold anti-fade with DAPI, P36931, Life Technologies, Thermo Fisher Scientific, Waltham, MA, USA) and then stored at −20°C until analysis. For immunofluorescence experiments, we used all normoxic (*n* = 25) and hypoxic animals (*n* = 25).

### Tyramide Signal Amplification for Detection of Complement Factor C5b-9

Due to the low signals using the C5b-9 direct immunofluorescence protocols, a tyramide signal amplification (TSA) procedure was employed. The TSA method is based on horseradish peroxidase (HRP)-conjugated secondary antibodies followed by incubation with a biotinyl tyramide-cyanine 5 conjugate (Perkin-Elmer, Waltham, MA, USA) diluted 1:150 in amplification buffer for 15 min at room temperature. Lastly, sections were washed three times for 15 min each in TBS-Tween 20 (0.05%) and then mounted using the standard protocol.

### Immunofluorescence – Analysis

Fluorescent images were obtained using a “VSlide” slide scanning microscope (MetaSystems, Alltlussheim, Germany). The system has a CoolCube 2 camera (12 bit gray scale), a 10× objective and filter sets for 4′,6-diamidino-2-phenylindole (DAPI) (EX350/50–EM470/40), Fluorescein isothiocyanate (FITC) (EX493/16−EM527/30), Cyanine (Cy) 3 (EX546/10−EM580/30), Cy3.5 (EX581/10−EM617/40), and Cy5 (EX630/20−647/long pass). First, the whole brain section was initially pre-scanned at 2.5× to adjust for focusing and generate a tissue-map. Tissue and focus depth were detected based on the DAPI signal. All tissue-covered areas were scanned using 10× objective. Finally, the individual images were stitched together (VSlide) to generate a large image of the entire section. After scanning, the images (vsi-files) were extracted to high quality Joint Photographic Experts Group (jpeg)-files for further analysis using the software Metaviewer^®^ (Metasystems, Alltlussheim, Germany). In order to facilitate the analysis, the images were downsampled 0.5× to Tagged Image File (tif)-files without losing any valuable biological information. An example of a scanned slide is available in Supplementary Material.

### Quantification of Immunofluorescence Staining

**Figure 2:**
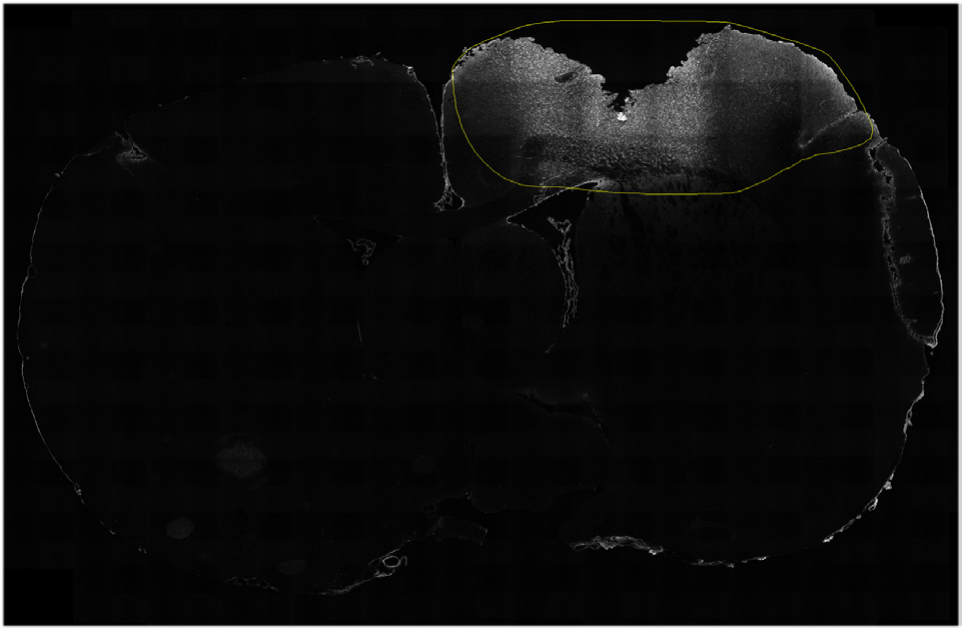
**Detection of expression**. A person blinded for oxygenation state and survival time highlighted the region of interest surrounding the lesion (yellow circle). The protein expression, above a specific threshold, was then used to quantify the expression. In this case, the expression of C5b-9 (white) is shown in a hypoxic animal, at 1 day following CCI.

**Figure 3:**
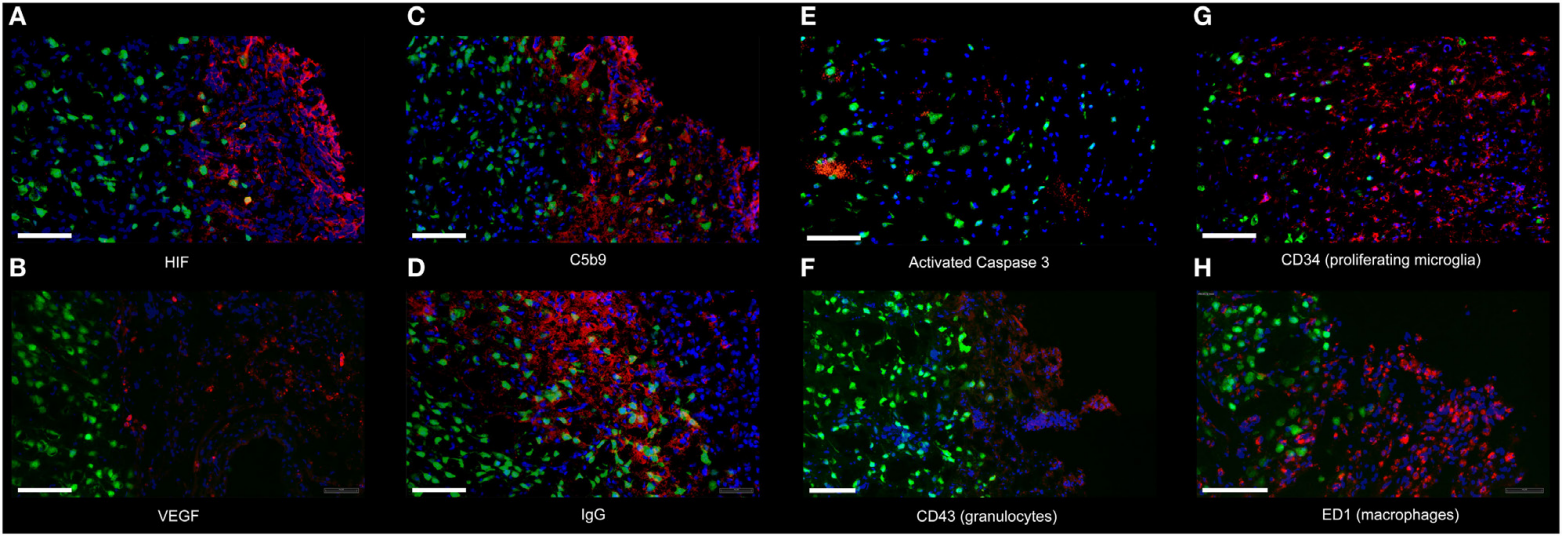
**Immunofluorescence of the analyzed proteins**. The image illustrates representative immunofluorescence to the following proteins: **(A)** HIF-1α (HIF) (day 1), **(B)** VEGF (day 1), **(C)** C5b-9 (day 1), **(D)** IgG (day 1), **(E)** Activated Caspase 3 (day 1), **(F)** Granulocytes (CD43, day 1), **(G)** Proliferating microglia (CD34, day 7), and **(H)** Macrophages (ED1, day 7) (all red, respectively) at 10× amplification from normoxic animals. NeuN was used to label neurons (green). DAPI was used as counterstaining for all cells (blue). All pictures are taken from the peri-lesional area; the cavity is located to the right of each picture. Scale bar = 100 μm.

ImageJ was also used to analyze immunoreactivity in the cortical tissue surrounding the lesion. A cut-off threshold was chosen for each antibody. The threshold varied depending on the signal to noise ratio dependent on signal intensity of each antibody in order to eliminate potential background noise. A person, blinded to oxygenation group and survival time, determined and outlined the perilesional area by mapping region of interests (ROIs) in ImageJ (Figure 2). These areas were then quantified in ImageJ, retaining the “integrated density” of the immunoreactivity (all pixels above the specified cut-off threshold), similar to methods previously described (62, 63). All available slides were used, with a minimum of three sections per brain for each antibody, only a few sections did not present with adequate quality and where omitted (see Table S1 in Supplementary Material). The set antibodies analyzed are illustrated in Figure 3. One rat (ET012, hypoxia 7 days) presented as an outlier for all antibody expression detected in the immunofluorescence experiments and was thus excluded from the study. Since no cavity was found in the sham animals, no immunofluorescence was quantified.

### NeuN and DAPI Immunofluorescence and Measurement of the Lesion Area

**Figure 4:**
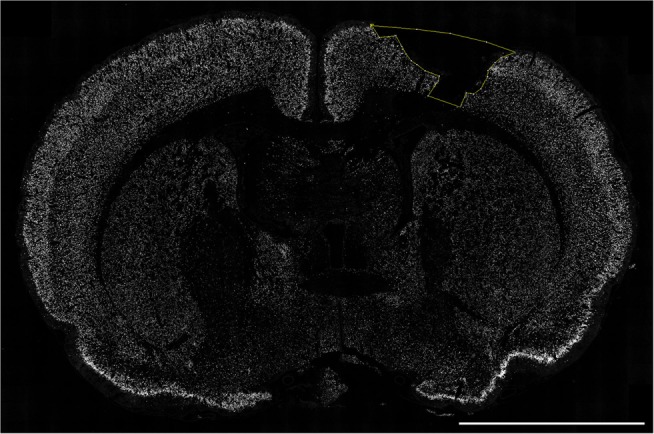
**Quantification of cortical neuronal cell death**. The section was labeled with NeuN (white) and the cortical lesion area (yellow) devoid of neurons was measured using the “Free hand” tool in ImageJ. Scale bar = 5 mm.

The software ImageJ was used to map the cortical layer devoid of NeuN-positive neurons, using the “Polygon selections,” also blinded for oxygenation and time after injury (Figure 4). DAPI was quantified using a similar approach. For analysis, we used at least three sections per brain to detect the largest lesion area, maximal area of neuronal loss in the cortex (NeuN), and general cellular/tissue loss (DAPI). No cavity was detected in sham animals and these were thus not included.

### Statistical Analysis

To correlate intra-operative monitored data and post-surgery metabolic data, Mann–Whitney *U* tests were employed to allow comparisons between normoxic and hypoxic animals. The MRI lesion size was illustrated by using bar plots and compared using a Mann–Whitney *U* test (GraphPad Prism 5.0, GraphPad Software, Inc., La Jolla, CA, USA).

A Student’s *t-*test was applied for S100B data to compare hypoxic and normoxic animals on the first day post-TBI as the data were normally distributed and this is the only time point were S100B could theoretically differ between the two groups since its serum half-life has been shown to be as short as 25 min (64).

Magnetic resonance imaging and immunoflourescence outcomes were assessed with a multiple linear regression model with oxygenation state and survival time as independent predictors. Oxygenation state (normoxia/hypoxia) was coded as a dummy variable. The logarithm of time was used as a continuous variable. This approach was chosen over ANOVA to better account for temporal trajectory as well as a global difference over time points between oxygenation state. The multiple linear regression model was visualized using ggplot2 (65) in R (66). More detailed information on the linear regression models used in this study is found in Table S2 in Supplementary Material.

An open Shiny (67) platform including our raw data is provided, where different analyses may be performed (https://thelin.shinyapps.io/Hypoxic-TBI-Frontiers).

A *p*-value <0.05 was considered statistically significant.

## Results

### Pre-Operative Monitoring and Post-Surgery Metabolic Data Confirm a Hypoxic Status

Physiological data revealed that the hypoxic animals had significantly lower oxygen saturation (*p* < 0.0001), pulse (*p* = 0.0314), and perfusion levels (<0.0001) compared to normoxic rats. Analysis of arterial blood samples demonstrated that these treatment groups presented significantly different metabolic conditions after 30 min of either normoxia or hypoxia. Levels of lactate (*p* < 0.0001), glucose (*p* = 0.0008), and pH (*p* = 0.0030) were significantly higher in hypoxic compared to normoxic animals while pCO_2_ (*p* < 0.0001) and pO_2_ (*p* < 0.0001) levels were significantly lower after hypoxia (Table 2). No difference was detected in arterial gas levels of hemoglobin, sodium, and potassium (Table 2). Some samples were not analyzed due to low blood volumes obtained or other technical difficulties (Table 2).

**Table 2:** **Post- and pre-operative monitoring parameters**.

	Normoxia	Hypoxia	*p*-Value	*n*
**Metabolic parameters (after 30 min normoxia/hypoxia)**				
Lactate (mmol/L)	**1.50 (1.20–1.75)**	**4.80 (3.38–6.56)**	**<0.0001**	**21 vs. 26**
Glucose (mmol/L)	**8.0 (7.0–8.5)**	**9.6 (8.0–12.0)**	**0.0008**	**21 vs. 25**
pCO_2_ (kPa)	**4.41 (3.85–5.75)**	**3.2 (2.57–3.68)**	**<0.0001**	**21 vs. 24**
pO_2_ (kPa)	**15.0 (10.4–17.3)**	**5.4 (4.9–5.7)**	**<0.0001**	**17 vs. 20**
pH	**7.41 (7.36–7.46)**	**7.49 (7.43–7.54)**	**0.0030**	**21 vs. 25**
Hemoglobin (g/dL)	125 (116–132)	127 (122–142)	0.3004	12 vs. 19
Na (mmol/L)	142 (139–147)	141 (139–143)	0.4211	21 vs. 26
K (mmol/L)	5.1 (4.0–6.4)	5.7 (4.4–7.9)	0.2223	21 vs. 26
**Monitored parameters (during 30 min normoxia/hypoxia)**				
Saturation (%)	**88 (87–90)**	**57 (55–61)**	**<0.0001**	**33 vs. 37**
Pulse (bpm)	**369 (351–399)**	**389 (365–428)**	**0.0314**	**33 vs. 37**
Perfusion (rate)	**0.18 (0.15–0.25)**	**0.11 (0.07–0.17)**	**<0.0001**	**33 vs. 37**

*Monitored physiological and metabolic parameters in normoxic vs. hypoxic animals. Mann–Whitney *U* Test was used to perform the statistical analysis. The varying degree of sample size was due primarily to loss of blood gas samples (too low levels) or other technical errors with the blood gas device (air bubbles in syringe). Bold indicates significance. Bpm, beats per minute*.

### Hypoxia Enhanced the Lesion Size and Neuronal Death after TBI

**Figure 5:**
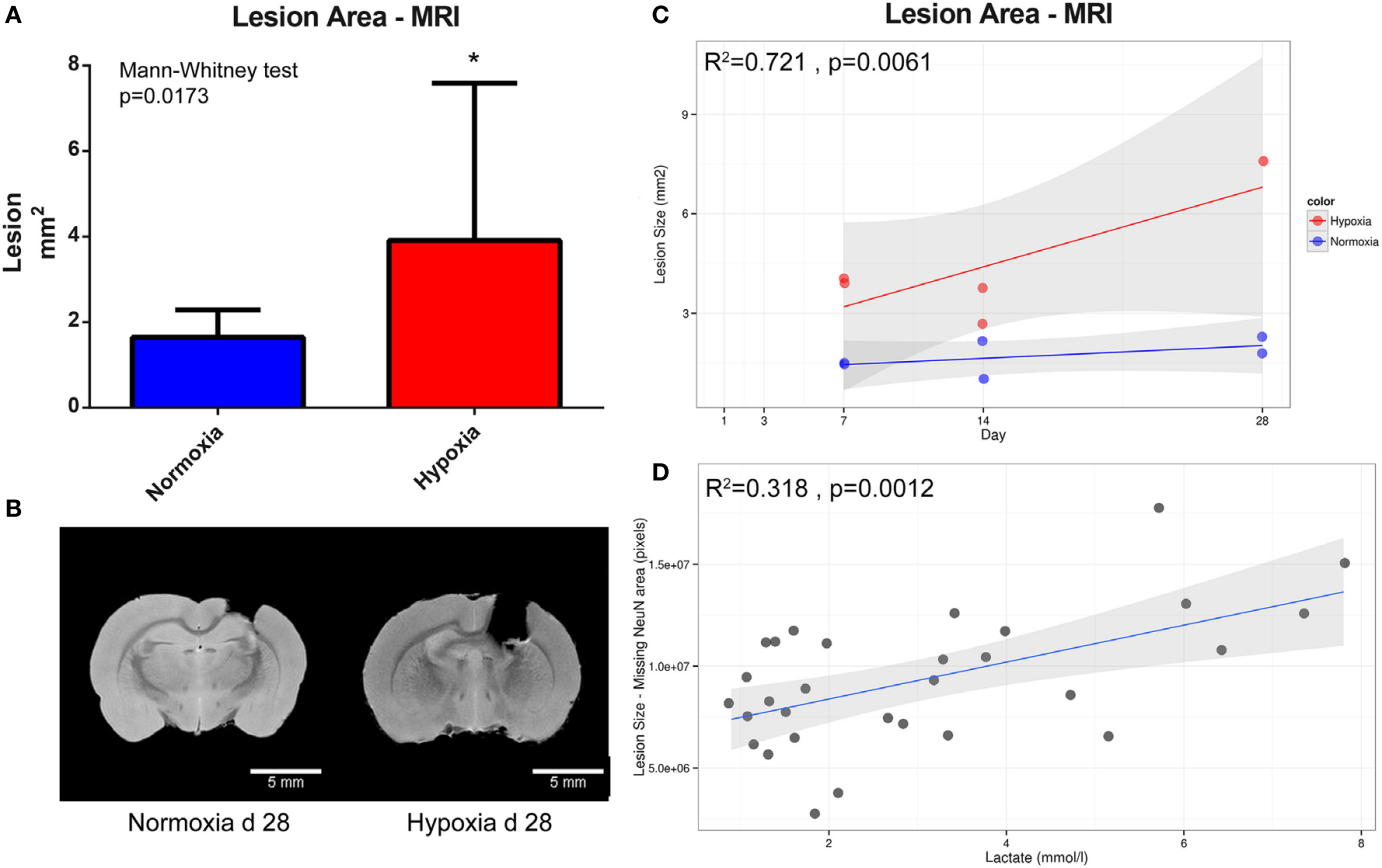
**Lesion size quantification**. **(A)** illustrates the difference in lesion size between the normoxic (*n* = 6) and hypoxic group (*n* = 5) at day 7, 14, and 28 post-CCI presented as mean and SD. In **(B)**, the lesion size on two representative animals is presented at day 28 following TBI; normoxia (left) and hypoxia (right). Scale bar = 5 mm. **(C)** illustrates changes in lesion size on MRI (*y*-axis, mm^2^) over time (*x*-axis, survival time in days) in hypoxic (red) and normoxic (blue) rats (*R*^2^ = 0.721). The gray area represents 95% confidence interval while the line is a regression line. **(D)** Correlations between the neuronal death using tissue area devoid of NeuN staining (*y*-axis, pixels) and all available lactate levels (*x*-axis, mmol/l) acquired from blood gas post surgery; as lactate increases, so does the lesion size (*R*^2^ = 0.318).

**Figure 6:**
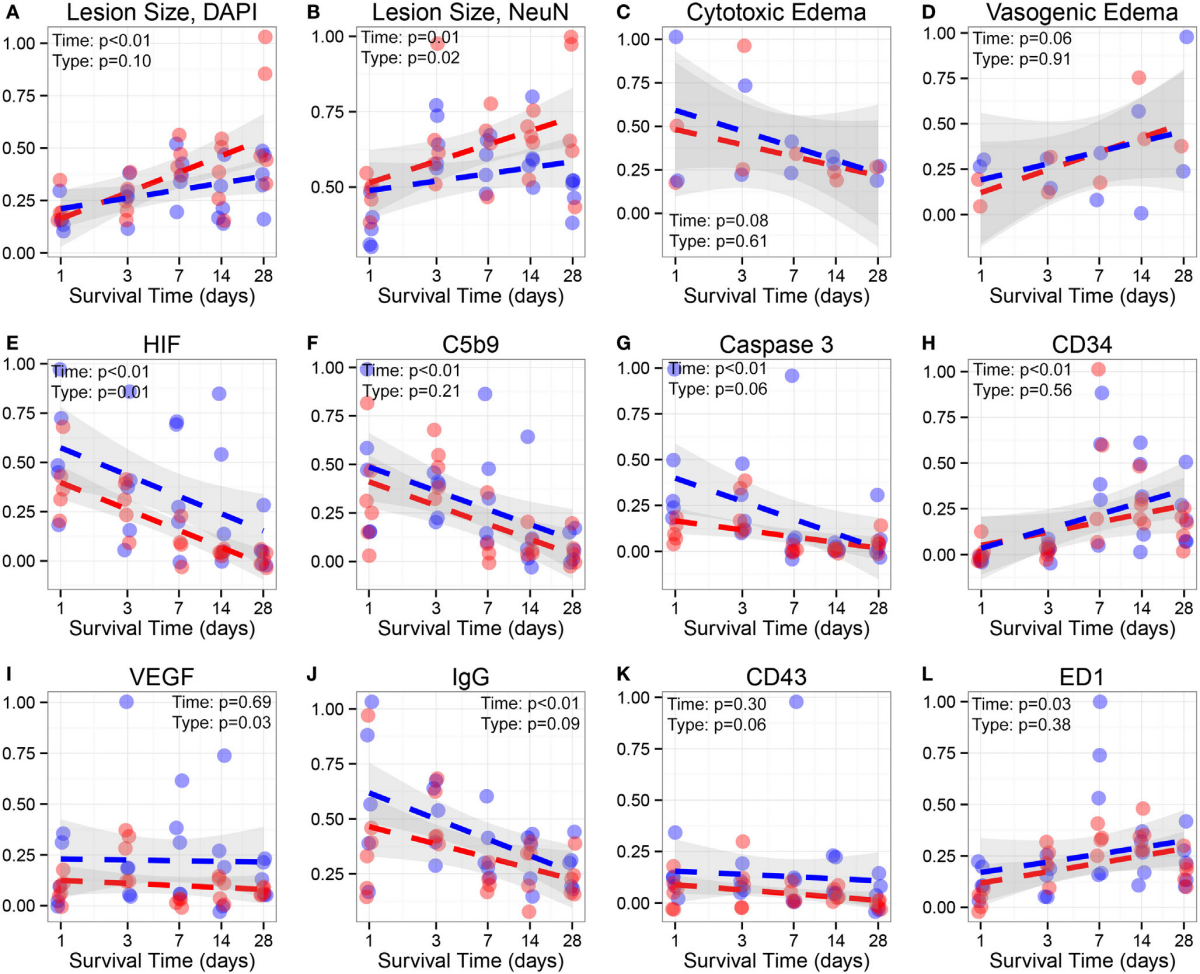
**Analyzes of lesion size, edema and immunofluorescence**. Lesion size, edema, and immunofluorescence data analysis in hypoxic and normoxic rats: **(A)**, Lesion size using DAPI staining, **(B)** Lesion size using NeuN staining, **(C)** Cytotoxic edema on MRI, **(D)** Vasogenic edema on MRI, **(E)** HIF-1α (HIF), **(F)** C5b-9, **(G)** Activated caspase-3, **(H)** CD34, **(I)** VEGF, **(J)** IgG, **(K)** CD43, and **(L)** ED-1 immunofluorescence intensity. *Y*-axis represent relative intensity. *X*-axis represents days after injury (log). Blue dots indicate normoxic while red dots represent hypoxic rats; dotted lines delineate regression lines. The gray area surrounding each line is the 95% confidence interval. Lesion area using DAPI and NeuN, HIF, C5b-9, Caspase-3, CD34, IgG, and ED1 changed significantly over time. Lesion Size, NeuN (*p* = 0.02), HIF (*p* = 0.01), and VEGF (*p* = 0.03) are significantly different between normoxic/hypoxic groups.

Examination of T2 MRI scans revealed that hypoxic animals had significantly larger lesion volumes compared to normoxic animals when later time points were combined for analysis (7, 14, and 28 days after injury) (*p* = 0.0173) (Figure 5A). Figure 5B depicts two representative brains harvested 28 days post-TBI in normoxic (left) and hypoxic (right) rats, with evident, significant difference in the lesion size between the two treatments. Figure 5C displays the increase of lesion size in hypoxic and normoxic rats measured on MRI over 4 weeks. The explanatory *R*^2^ containing both oxygenation (hypoxia or normoxia) level and survival time was 0.721. Moreover, a correlation between higher blood lactate levels and increasing area of neuronal death, based on NeuN staining, in all animals with lactate levels was detected (*p* = 0.0012) (Figure 5D). Based on NeuN staining, we detected a significantly increased rate of neuronal loss in rats exposed to a hypoxic insult compared to normoxic animals (*p* = 0.0316) (Figure 6). Consistent with this finding, the lesion size measured in hypoxic animals using DAPI staining also increased over time, albeit without reaching statistical significance (*p* = 0.10) (Figure 6).

### Cytotoxic and Vascular Edema Do Not Differ in Hypoxic and Normoxic Rats

Based on MRI analysis, we aimed at distinguishing the temporal patterns of cytotoxic and vasogenic edema in hypoxic and normoxic animal groups (Figure 6). Cytotoxic edema decreased rapidly over time while the area obtained for vasogenic edema increased, although not significantly. When comparing both treatment groups, we found no differences in the extent of either form of brain edema.

### Serum Levels of S100B Tended to Be Higher in Hypoxic Rats at 1 Day Post-TBI

**Figure 7:**
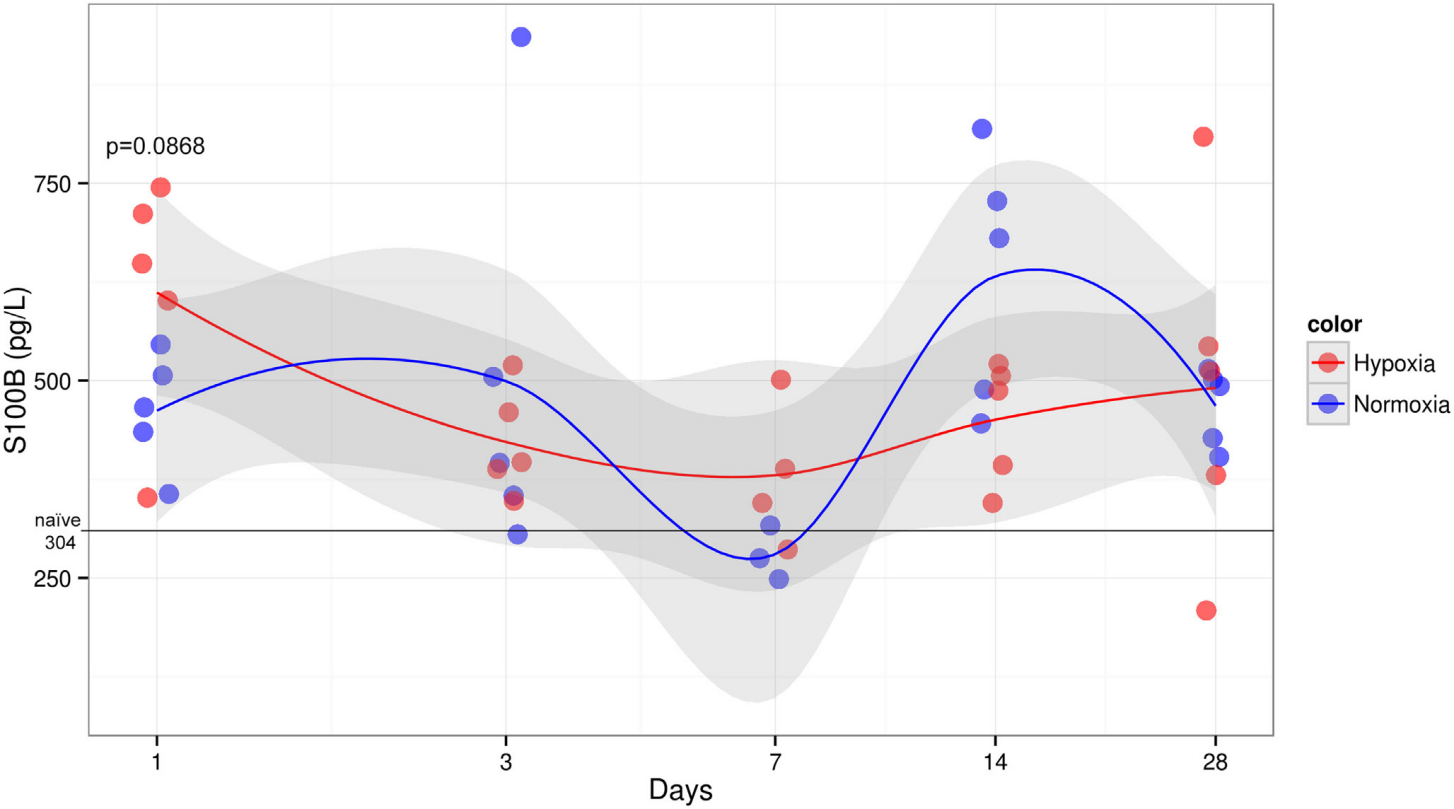
**Serum S100B levels over time after injury**. Serum concentration of S100B (*y*-axis) measured at different times post-CCI (*x*-axis). Black line indicates median naive levels. S100B shows a trend to be significantly elevated in hypoxic compared to normoxic group at 1 day after injury (Student’s *t*-test *p* = 0.0868). No other survival time showed any significance or trend between groups, yet levels remained elevated compared to naive rats. No significant temporal trend could be detected.

The concentration of S100B in hypoxic animals at 1 day post-injury was slightly higher when compared to normoxic counterpart showing a trend toward a significant difference (*p* = 0.0868) (Figure 7). At later time points following TBI, both treatment groups failed to show any significant differences in S100B, which, however, remained elevated compared to naive animals that presented with low S100B levels (median 304 pg/l). Sham animals also presented elevated S100B levels, especially hypoxic animals 24 h after injury (median 773 pg/l) (data not shown). Three animals (two normoxic on day 7 and one hypoxic sham on day 1) had levels higher than 5 SD and were excluded from the analysis.

### HIF-1α and VEGF Are More Elevated in Normoxic Rats

Hypoxia-inducible factor-1 alpha and VEGF expression was higher in normoxic animals compared to hypoxic animals, especially at the later time points (7, 14, and 28 days) (*p* < 0.05, respectively) (Figure 6). Following an initial upregulation, HIF-1α decreased significantly over time while VEGF expression remained unchanged.

### Activated Caspase 3 and C5b9 Expression Decreases Over Time but Is Not Significantly Different between Hypoxic and Normoxic Animals

While activated caspase 3, indicating ongoing apoptosis, was higher in the first 3 days after TBI, there was no difference between the oxygenation groups or at any time over the 4 weeks. C5b9 was found upregulated in the perilesional area, primarily in neurons surrounding the contusion. The highest levels were seen immediately after trauma and then steadily decreased over time (Figure 6). No significant differences were detected between oxygenation groups.

### Immune Cell Activation Is Not Affected by Hypoxia

ED1 (predominantly present in macrophages) and CD34 (predominantly in microglia) expressions were predominantly seen in the perilesional subcortical white matter, although also spread out over the perilesional cortical areas (Figure 6). CD34 does not only stain microglia but is also seen in stem cells in vascular tissue (displayed as blood vessels); however, microglia-like morphology was easy to distinguish (58) and was more highly expressed than the vasculature. ED1 morphology was different than CD34, as is seen in Figure 4, but the antibody stains activated microglia as well making it difficult to separate the two (and they share expression as is seen in Figure 6). CD43 (granulocyte) expression was only detected in cells the perilesional area. ED1 and CD34 expression revealed a temporal pattern, increasing significantly over time while CD43 remained unchanged.

### Blood–Brain Barrier Damage Increases Acutely after TBI but Is Not Different between Hypoxic and Normoxic TBI Rats

IgG extravasation, as a sign of BBB disintegration, was evident in the perilesional cortex of both oxygenation groups and peaked early after injury to then steadily decrease over time. No significant difference in the amount of IgG extravasation was found between hypoxic and normoxic animals (Figure 6). Notably, the intensity of IgG immunoreactivity was highly correlated with complement activation based on C5b9 immunofluorescence (*R*^2^ = 0.759), implying a pathophysiological relationship.

## Discussion

In this study, we demonstrated that a clinically validated post-traumatic hypoxia paradigm significantly exacerbates the lesion size and neuronal loss following CCI in rats. S100B levels in serum had a tendency to be higher in hypoxic rats 24 h after trauma. HIF-1α and VEGF immunofluorescence was significantly increased only in normoxic but not in hypoxic rats, perhaps highlighting their beneficial properties toward surviving neurons located in the perilesional area of animals presenting a less severe brain damage without hypoxia. To the best of our knowledge, this is the first report that investigates how general hypoxia affects the inflammatory response in a CCI model.

In our study, a rise in blood lactate levels, as a surrogate marker of hypoxia, was significantly correlated to diminished neuronal survival. The analysis of lactate levels relative to survival times provided a strong (*R*^2^ 0.47%) explained variance of neuronal survival. We believe that lactate is a more reliable indicator of hypoxia than pO_2_ or SO_2_ since gas changes are more volatile and more easily influenced by venous blood contamination. Glucose was also significantly higher in the hypoxic rats, perhaps as sign of ongoing metabolic distress and subsequent catecholamine surge, both of which have shown to affect outcome in TBI patients (68). These metabolic changes are also supported by a decrease in perfusion index in hypoxic rats, indicating peripheral vasoconstriction. In summary, we demonstrated that hypoxia following focal TBI has detrimental effects on neuronal survival, being directly correlated to enhanced levels of lactate immediately after injury.

Using histopathological examination, other groups have shown that the lesion in focal TBI models is amplified following hypoxia (17, 45). The loss of cortical neurons was higher in hypoxic rats, which is supported by previous findings in a CCI model of hypoxic TBI (46). The incidence of neuronal death has been shown to increase during the first week following a focal hypoxic-TBI, especially in the hippocampus (22, 23, 45, 46, 69), likely involving a population of neurons that are more susceptible to metabolic distress. In our study the lesion area devoid of nuclear DAPI signal was not statistically different in hypoxic or normoxic animals, but only showed a trend toward larger lesion after hypoxia (*p* = 0.10). However, the lesion area observed with DAPI often presented abundant tissue debris in the lesion cavity, especially during the early times following CCI; a fact that made it unreliable for lesion quantification. Consequently, more emphasis was given to lesion measurement using NeuN stained sections. While other studies have used MRI to monitor only the extent of edema formation acutely following experimental hypoxic-TBI (19, 20, 47), in our study, we employed the MRI technology to combine measurements of edema with lesion size over 4 weeks and demonstrated that the lesion size increased over time, especially in hypoxic rats. Furthermore, the finding of a larger lesion by MRI in hypoxic rats is corroborated by a significantly greater contusion size on brain sections stained with NeuN. While early after CCI, the core of the lesion becomes necrotic, over time the surrounding uninjured tissue will degenerate due to the progression of edema, activated neurotoxic cascades and cell loss (70). In regard to the extent of edema, we did not find a significant difference between normoxic and hypoxic rats. This finding is similar to a study by a collaborating group that failed to detect any significant increase of brain edema in hypoxic TBI rats compared to TBI alone (13). Other studies reporting a significant increase in edema in hypoxic animals, compared to normoxic, used fluid percussion models to inflict TBI (19, 20, 47, 71), which provides a more well defined injury area not effected by a lesion cavity, presumably better for quantifying edema formation. Both cytotoxic and vasogenic edema have been shown play an important role if a hypoxic insult is added to brain trauma (18, 20). In CCI models, cytotoxic edema has been suggested to be the prominent type of edema occurring in the tissue surrounding the lesion (56, 70) as confirmed in our *ex vivo* MRI experiments. We observed an initial increase of cytotoxic edema followed by a gradual decrease; a result in line with previous studies showing low levels of cytotoxic edema after 7 days (72, 73). Similar to our findings, these reports demonstrated that vasogenic edema was localized in close proximity to the lesion (54, 74). The amount of vasogenic edema increased over time albeit not significantly, probably due to the small sample size of the animal groups. This finding is similar to another study of hypoxic TBI that revealed an initial increase of cytotoxic edema that switched into vasogenic edema later on (47). In conclusion, we report here that rats subjected to post-traumatic hypoxia presented significantly increased lesion areas using immunohistochemistry and MRI compared to normoxic animals while no significant difference in either cytotoxic or vasogenic edema could be detected between the groups using our model of focal hypoxic TBI.

The levels of S100B were higher in hypoxic rats 1 day after injury, compared to normoxic rats, albeit not reaching significance (*p* = 0.0868). The lack of difference is presumably an effect of low power due to a small sample size. For the later survival times, no difference between the oxygenation groups was observed, which is probably the result of the limited half-life of S100B being as short as 25 min in humans (64). Our experience in human studies indicates that S100B peaks early after TBI and gradually decreases in the acute phase. However, the levels of S100B remained increased compared to naive rats, which could be due to ongoing gliosis that has been shown to persist for at least 4 weeks following TBI (75), which might present both beneficial and detrimental effects. Furthermore, on day 1 the hypoxic sham animals had similar levels of S100B as the hypoxic TBI animals, suggesting that the hypoxia insult alone and the craniotomy are associated with increased S100B levels. Osteocytes and dermal cells express S100B (76) and could potentially be a source to S100B increases early after injury (77). Earlier (presumably only hours after TBI) sampling is probably necessary to determine the potential of S100B as a biomarker of injury severity. In TBI patients, S100B has been shown to correlate to the extent of hypodense lesions at hospital admission, perinatal asphyxia as well as subsequent ischemic development (40, 42, 78), indicating that if ischemia is added to brain injury, it would lead to an additional increase of S100B. Altogether, our and other studies support the validity of S100B measured early after trauma as a potential biomarker to monitoring severe hypoxic injuries following TBI, albeit further investigations including earlier survival times are warranted to corroborate this assumption.

In our study, we also demonstrated that normoxic animals had higher expression of HIF-1α, which was more obvious at later time points after CCI (7–14 days). While upregulation of HIF-1α has been used to validate hypoxia–ischemic brain injury in neonatal rats (79), HIF-1α has, to our knowledge, never before been used to validate hypoxic insults following experimental hypoxic-TBI. However, HIF-1α has been previously analyzed under normoxic conditions in experimental TBI where it has revealed a temporal pattern with an early increase (1 h up to 3 days) followed by a later decrease in expression (60, 80, 81), which was correlated with the degree of cell apoptosis. In our study, a similar temporal pattern of HIF-1α was noted. Previous work has revealed that an upregulation of HIF-1α does not necessarily mean that the injured tissue will perish. Although it is recognized that HIF-1α promotes apoptotic neuronal death (30), it is also known that it upregulates angiogenic factors (VEGF), as well as erythropoiesis and acts protectively against mitochondrial and cellular damage (32, 35, 36, 82). Perhaps in our hypoxic animals, the perilesional tissue succumbed rapidly after injury due to necrosis, as supported by a greater neuronal loss, while in normoxic animals, the tissue comprising the penumbra survived, hence being able to express HIF-1α. This somewhat paradoxical finding is supported by reviews suggesting that in milder hypoxic states, HIF-1α triggers neuro-protective mechanisms, while in more severe hypoxia, apoptosis and cellular death often follows HIF-1α activation; thus there is a distinction between mild and severe hypoxia inducing protective gene expression mediated by HIF-1α in the first instance and apoptotic gene expression in the severe form (30, 82). In addition, we showed that HIF-1α was strongly correlated with VEGF activation (data not shown), as reported earlier (32), highlighting the neuro-protective capabilities of HIF-1α. Similar to HIF-1α, VEGF expression was found to be upregulated primarily 3, 7, and 14 days following injury, being significantly higher in the normoxic group compared to the hypoxic group, yet without any significant change over time. Previous studies have shown a peak of VEGF expression 4–6 days following TBI (83), whereby VEGF seems to act as a promoter of neurogenesis after injury (83, 84). By increasing VEGF expression levels, the lesion size in experimental TBI has been shown to decrease, supporting its neuro-protective role (61). All in all, upregulation of HIF-1α and VEGF might reflect the neuro-protective capabilities of surviving cells, which could explain their increase following TBI during normoxic conditions.

Activated caspase-3 expression, is an indicator of ongoing apoptosis, which was elevated primarily in normoxic animals early after injury (up to day 3) but then relatively low in both hypoxic and normoxic animals. While apoptosis has been primarily identified in hippocampal cell layers following hypoxic TBI (23, 46), the amount of caspase-3 driven apoptosis in the cortical layers has never previously been shown in hypoxic TBI. Similar to other protein expressions levels, activated caspase-3 was more elevated in normoxic animals, presumably as hypoxic animals suffered more of immediate necrosis in the border zone following TBI whereas normoxic animals had more neurons that succumbed due to apoptosis. Microglia proliferation has been previously described in focal TBI within the surrounding contusional areas as well as in white matter regions in diffuse TBI (14, 27), with a peak after about 7 days and continuing for 4 weeks, similar to our findings. Akin to caspase 3-acitivation, pyramidal cell layers in cortical and hippocampal neurons appear to be particularly vulnerable after injury and hypoxia as microglial proliferation has been shown in these regions as well (85). Microglia activation was similar in localization and time to macrophage migration was not found to be significantly increased in hypoxic animals. The role of macrophages recruited to the injured brain has been suggested to include phagocytosis of dying cells as well as to remove tissue debris resulting from TBI (86). Previous studies have shown that macrophages start to migrate and accumulate in the perilesional area over 1–4 days after injury reaching a peak at 7 days after focal TBI models (27, 87), which was similar in our study. In a diffuse model of TBI, adding a similar hypoxic insult resulted in a macrophage infiltration restricted to brain areas with evident axonal pathology (corpus callosum, optic tract, and brain stem) being highest at 7 and 14 days in both hypoxic and normoxic groups (14), with significantly increased infiltration in corpus callosum of hypoxic animals. However, the models used are different (diffuse vs. CCI) making comparison of such studies difficult. Granulocyte accumulation in the perilesional zone, using the marker CD43, has been shown to be highest early (1–3 days) after injury (27, 50). However, in our model, we did not detect any changes for CD43 immunoreactivity in the study period, neither did we see any significant difference relative to hypoxic and normoxic treatments. Altogether, while we did not see a difference in activated caspase-3, microglial- (CD34), macrophage- (ED-1), and granulocyte- (CD43) infiltration between the different oxygenation states over 4 weeks, activated caspase-3 was significantly increased early after injury while microglia and macrophage activity increased later on.

In a fluid percussion model of focal hypoxic TBI, BBB disintegration has been demonstrated *via* increased amount of intracerebral IgG (18). In our animals, we saw a peak of IgG extravasation early after injury, which is supported by previous reports of increased BBB permeability 1–3 days following TBI (88–90). Furthermore, IgG auto-antibodies have also been detected, binding to perilesional neurons following focal TBI, which has been suggested to be involved in phagocytosis of these cells (91). In line with the edema results from the MRI, which would indicate BBB disturbance, we could not detect a greater parenchymal IgG immunoreactivity in the hypoxic group. Complement activation has been reported to play an important role following TBI (27, 28, 57), with its upregulation leading to an increase in cellular death, primarily involving neurons, in the perilesional area (92). To our knowledge, this is the first study to examine complement activation and specifically the end-stage product, the membrane attack complex C5b-9, in a hypoxic TBI model. We detected the same temporal pattern that has been previously described for C5b-9 expression (57), being significantly elevated 1–3 days compared to 14 and 28 days after CCI, especially in proximity of neurons in the perilesional area. However, C5b-9 upregulation did not differ in hypoxic compared to normoxic animals. Interestingly, increased C5b-9 correlated to IgG extravasation and cytotoxic edema. Previous clinical studies have suggested that BBB breakdown and subsequent increased vascular permeability is attributed to complement activation, which is further supported by our current findings (93, 94). Perhaps the lack of differences is due to the choice of relatively late time points after CCI (≥24 h) thus missing the marked disintegration of the BBB, which is known to occur in the early hours following TBI (88). In summary, C5b-9 expression and cerebral IgG extravasation were not significantly exacerbated or increased in hypoxic TBI but were amplified as a result of TBI in normoxic animals.

In summary, while we believe that our choice of CCI as injury mechanism better mimics a human focal injury, it is more difficult to measure the surrounding border zone of injured tissue due to the heterogeneous lesion cavity, presumably making other methods of more diffuse injury (such as fluid percussion- or diffuse axonal injury models) better to quantify pathophysiology in the affected tissue as these will not create widespread necrosis in the brain parenchyma. In general, immunohistochemical detection of a number of factors in the perilesional zone was more elevated in normoxic rats compared to hypoxic, which we believe is due to a greater extent of surviving tissue in these animals as they present with smaller lesion cavities. Moreover, we noted that the expression of several tissue proteins and blood S100B levels were similar between the groups, which could be the effect of too late time-points. We believe this is the case especially for S100B, cytotoxic edema, IgG and C5b-9 expression, which should probably be measured hours after injury to detect potential differences between the oxygenation groups. Difficulties also emerges when human studies are compared with rodent animal models, since rats theoretically have a faster protein metabolism than humans and one rat day represents about 90 human days (95, 96). However, while this study lacks early time points, it is a unique example of well monitored animals with longer survival times, followed with MRI examinations up to 4 weeks after injury, indicating a distinct and well-described lesion development and with potential neuro-protective mechanisms developing in primarily animals that suffer TBI during normoxic conditions.

### Limitations

In previous studies analyzing protein expression, edema and inflammation following experimental hypoxic TBI, a range of *n* = 3 up to *n* = 10 animals per group has been used (16, 20, 44–46, 69). We used *n* = 5 animals per group per time-point which we believe is enough to be able to show a difference in our immunohistochemistry quantifications, based on previous studies. Even if we only performed *n* = 2 MRI scans per group per time-point, due to limited resources, we aggregated the samples using our multiple linear regression models for edema (*n* = 10 vs. *n* = 9) and used a Mann–Whitney *U* test for lesion size (*n* = 6 vs. *n* = 5), hence increasing power. Moreover, we never draw any independent conclusions from the MRI analyses, instead we combine them with other outcome metrics to tell the full story of the pathophysiological mechanism that we believe occurred following hypoxic TBI in a CCI model.

Although all animals included in the hypoxic group had lactate levels greater than the normoxic counterparts, the variability within the hypoxic group was quite large, indicating that different biological responses to a varying degree of pO_2_ may be possible. Moreover, rats with low pCO_2_ had higher lactate levels, probably as a compensatory mechanism (Figure S3 in Supplementary Material). Other studies also reported of lower pCO_2_ in hypoxic-TBI animals (14, 21, 97), albeit not significantly lower than normoxic rats, which could be an effect of lower sample sizes compared to this study. However, low pCO_2_ is presumably occurring in humans suffering from metabolic acidosis due to hypoxia post TBI and the corresponding attempt to hyperventilate. This could also be an explanation for the paradoxically elevated pH in the hypoxic animals. If muscle relaxation had been provided in our study, it would perhaps have generated a more homogenous hypoxic group as ventilation could be better controlled for. This finding further stresses the need for adequate monitoring of animals, something which is regrettably lacking in many studies of experimental hypoxic-TBI.

Unfortunately, not all rats had blood samples drawn for gas analysis after surgery. Low blood volumes, technical difficulties, and procedural complications resulted in obtaining blood gas samples from 67% of the animals. While this may not a major limitation for group comparisons, as the rats without blood drawn presented with similar saturation, pulse and perfusion levels as the rats with blood gas measurements, we do believe that a larger sample size of rats with lactate measurements would have provided a better ability to correlate S100B, immunohistochemistry expression, and MRI measurements with the severity of the hypoxic event.

Our choice of using immunohistochemistry to quantify the expression of different proteins in brain parenchyma is associated with several limitations. One is that only a two-dimensional area is quantified. Western blot still remains a golden standard to determine protein expression in experimental TBI experiments. However, it lacks locational and anatomical information of protein expression, and several of the quantified proteins are expressed in uninjured areas of the brain as well. Our model includes only protein expression within the border zone of the injury, which is the main ROI in our study. In order to include largest lesion volumes, we used several brain sections to find the maximal expression, therefore, we believe that we have a more reliable measurements of true perilesional protein expression compared to Western blot.

As previously discussed, ED-1 could stain both microglia and macrophages, and CD34 was not only increased in proliferating microglia but also in vascular stem cells. However, through morphology, we could detect what we believe represents true representations of macrophages and microglia and by using adequate cut-off thresholds for quantification, only true expression was used in the analyses. CD43 is also present in a variety of leukocytic cells, perhaps not only indicating granulocyte infiltration in the perilesional zone but also other cells of hemopoietic origin.

All previous experimental studies on post-TBI hypoxia included male SD rats, while we used female SD rats. We did not aim to analyze pathologic difference between the sexes, but used female rats for practical reasons as more easily available in our facility. Female sex hormones, especially progesterone, have been extensively studied as a neuro-protective agent (98–100). While neuronal death (101) and neuroinflammation (102) decreased in brain-injured rats treated with progesterone, cytotoxic edema was not altered by progesterone therapy (103) In addition, when male and female rats are compared, assessing COX-2 response in penetrating TBI, differences are small (104). In this context, the topic of gender and differences in TBI remains controversial. In fact, in human TBI, the female gender seems to be a negative predictor of outcome (105), even if protective effects of the lutheal phase in the menstrual cycle have been shown in mild TBI (106). Surprisingly, despite the experimental evidence, the beneficial properties of progesterone therapy were not substantiated in a human clinical trial, ProTECT III that was abandoned due to futility (107). Altogether, while the choice of female rats might have impaired the opportunity to compare our study to others based on male rats, it is difficult to determine to what extent, as often similar studies on male rats presented striking differences in outcome measures. It is clear that further work is warranted to fully elucidate differences between genders in terms of pathologic and neurologic outcomes after TBI.

## Conclusion

Our study has shown that adding a hypoxic insult to a model of TBI using the CCI paradigm, lead to increased neuronal loss and lesion size with a trend toward higher levels of the biomarker S100B in serum 1 day after trauma compared to normoxic animals. HIF-1α and VEGF were found more elevated in normoxic TBI, primarily at later survival times, perhaps indicating ongoing neuro-protective mechanisms. Further studies are warranted to fully determine the pathophysiological effect of hypoxia following TBI.

## Author Contributions

ET, AF, B-MB, MR, MS, and MM-K designed and planned the study. ET and AF conducted the animal experiments with the help from MS, B-MB, MM-K, and MR. ET, PD, MR, and SA conducted the MRI examinations. ET and MR carried out the ELISA. ET, NM, and JM performed the immunohistochemistry, which was quantified by AF and ET, with antibodies supplied from B-MB, MR, and MS. ET and AF performed the statistical analyses. ET drafted the manuscript, which all authors read and approved.

## Conflict of Interest Statement

The authors declare that the research was conducted in the absence of any commercial or financial relationships that could be construed as a potential conflict of interest.
