# Supplementary material for: Lesion Size Is Exacerbated in Hypoxic Rats Whereas Hypoxia-Inducible Factor-1 Alpha and Vascular Endothelial Growth Factor Increase in Injured Normoxic Rats: A Prospective Cohort Study of Secondary Hypoxia in Focal Traumatic Brain Injury
Source: Front Neurol. 2016 Mar 7;7:23. doi: 10.3389/fneur.2016.00023 (PMC4780037; doi:10.3389/fneur.2016.00023)
Supplement: Supplementary file 1 [file Table_1.DOCX]

Supplementary Table 1

| Protein | n missing |
| --- | --- |
| IgG | 4/165 (2%) |
| C5b-9 | 4/165 (2%) |
| ED1 | 3/162 (2%) |
| CD43 | 3/165 (2%) |
| HIF1 | 8/162 (5%) |
| VEGF | 1/165 (1%) |
| Caspase3 | 4/162 (2%) |
| CD34 | 2/165 (1%) |

Supplementary Table 1. Missing slides from immunohistochemistry examination due to low quality because of tissue folding, out of focus etc. No more than 1 slide per brain was excluded, thus at least 2 slides per brain were always used to detect optimal expression.
