# Supplementary material for: Lesion Size Is Exacerbated in Hypoxic Rats Whereas Hypoxia-Inducible Factor-1 Alpha and Vascular Endothelial Growth Factor Increase in Injured Normoxic Rats: A Prospective Cohort Study of Secondary Hypoxia in Focal Traumatic Brain Injury
Source: Front Neurol. 2016 Mar 7;7:23. doi: 10.3389/fneur.2016.00023 (PMC4780037; doi:10.3389/fneur.2016.00023)
Supplement: Supplementary file 2 [file Table_2.DOCX]

Supplementary Table 2

| Expression of; | Estimate | Std. Error |  | T-value | | | R-square | | Adjusted R-squared | | F-statistic | | p-value |
| --- | --- | --- | --- | --- | --- | --- | --- | --- | --- | --- | --- | --- | --- |
| DAPI - Model |  |  | | |  |  | | 0.315 | | 0.286 | | 10.59 | 0.00016 |
| DAPI - Time | 2546952 | 593256 | | |  | 4.29 | |  | |  | |  | <0.01 |
| DAPI - Oxygenation | -2335696 | 1398322 | | |  | -1.67 | |  | |  | |  | 0.10 |
| Cytotoxic - Model |  |  | | |  |  | | 0.2 | | 0.093 | | 1.87 | 0.18799 |
| Cytotoxic - Time | -0.59 | 0.31 | | |  | -1.90 | |  | |  | |  | 0.08 |
| Cytotoxic - Oxygenation | 0.38 | 0.73 | | |  | 0.52 | |  | |  | |  | 0.61 |
| Vasogenic - Model |  |  | | |  |  | | 0.215 | | 0.111 | | 2.06 | 0.16226 |
| Vasogenic - Time | 0.35 | 0.18 | | |  | 2.01 | |  | |  | |  | 0.06 |
| Vasogenic - Oxygenation | 0.05 | 0.41 | | |  | 0.11 | |  | |  | |  | 0.91 |
| NeuN - Model |  |  | | |  |  | | 0.236 | | 0.203 | | 7.1 | 0.00205 |
| NeuN - Time | 961100 | 330315 | | |  | 2.91 | |  | |  | |  | 0.01 |
| NeuN - Oxygenation | -1871040 | 778563 | | |  | -2.40 | |  | |  | |  | 0.02 |
| HIF1A - Model |  |  | | |  |  | | 0.419 | | 0.394 | | 16.57 | <0.00001 |
| HIF1A - Time | -135389065 | 27199973 | | |  | -4.98 | |  | |  | |  | <0.01 |
| HIF1A - Oxygenation | 186254188 | 64111197 | | |  | 2.91 | |  | |  | |  | 0.01 |
| Caspase 3 - Model |  |  | | |  |  | | 0.253 | | 0.221 | | 7.81 | 0.0012 |
| Caspase 3 - Time | -44710547 | 12976190 | | |  | -3.45 | |  | |  | |  | <0.01 |
| Caspase 3 - Oxygenation | 59432427 | 30585290 | | |  | 1.94 | |  | |  | |  | 0.06 |
| C5b-9 - Model |  |  | | |  |  | | 0.317 | | 0.287 | | 10.88 | 0.00013 |
| C5b-9 - Time | -50765806 | 11217216 | | |  | -4.53 | |  | |  | |  | <0.01 |
| C5b-9 - Oxygenation | 34250049 | 26772049 | | |  | 1.28 | |  | |  | |  | 0.21 |
| ED1 - Model |  |  | | |  |  | | 0.441 | | 0.416 | | 18.11 | <0.00001 |
| ED1 - Time | 17675765 | 7808379 | | |  | 2.26 | |  | |  | |  | 0.03 |
| ED1 - Oxygenation | 16250013 | 18404596 | | |  | 0.88 | |  | |  | |  | 0.38 |
| VEGF - Model |  |  | | |  |  | | 0.097 | | 0.058 | | 2.52 | 0.09132 |
| VEGF - Time | -591305 | 1477005 | | |  | -0.40 | |  | |  | |  | 0.69 |
| VEGF - Oxygenation | 7828288 | 3525157 | | |  | 2.22 | |  | |  | |  | 0.03 |
| IgG - Model |  |  | | |  |  | | 0.29 | | 0.26 | | 9.6 | 0.00032 |
| IgG - Time | -21763322 | 5328638 | | |  | -4.08 | |  | |  | |  | <0.01 |
| IgG - Oxygenation | 21860807 | 12717823 | | |  | 1.72 | |  | |  | |  | 0.09 |
| CD43 - Model |  |  | | |  |  | | 0.092 | | 0.053 | | 2.38 | 0.1038 |
| CD43 - Time | -3388836 | 3206074 | | |  | -1.06 | |  | |  | |  | 0.30 |
| CD43 - Oxygenation | 14852519 | 7651914 | | |  | 1.94 | |  | |  | |  | 0.06 |
| CD34 – Model |  |  | | |  |  | | 0.171 | | 0.136 | | 4.86 | 0.01204 |
| CD34 – Time | 598817 | 196932 | | |  | 3.04 | |  | |  | |  | <0.01 |
| CD34 - Oxygenation | 278515 | 470015 | | |  | 0.59 | |  | |  | |  | 0.56 |

Supplementary Table 2. More detailed statistics for the linear models used. “Model” includes “oxygenation” and logged “days after injury” (time). “Time” illustrates how the protein expression changed over time. “Oxygenation” illustrates how protein expression changed between normoxia and hypoxia.
