# Supplementary material for: Lesion Size Is Exacerbated in Hypoxic Rats Whereas Hypoxia-Inducible Factor-1 Alpha and Vascular Endothelial Growth Factor Increase in Injured Normoxic Rats: A Prospective Cohort Study of Secondary Hypoxia in Focal Traumatic Brain Injury
Source: Front Neurol. 2016 Mar 7;7:23. doi: 10.3389/fneur.2016.00023 (PMC4780037; doi:10.3389/fneur.2016.00023)
Supplement: Supplementary file 4 [file Presentation_1.ZIP › index.html]

krpano.com - ET.Cover slide~F-Spot000001


|  |
| --- |
| ERROR:  Javascript not activated |
