# Supplementary figures and images for: Lesion Size Is Exacerbated in Hypoxic Rats Whereas Hypoxia-Inducible Factor-1 Alpha and Vascular Endothelial Growth Factor Increase in Injured Normoxic Rats: A Prospective Cohort Study of Secondary Hypoxia in Focal Traumatic Brain Injury
Source: Front Neurol. 2016 Mar 7;7:23. doi: 10.3389/fneur.2016.00023 (PMC4780037; doi:10.3389/fneur.2016.00023)

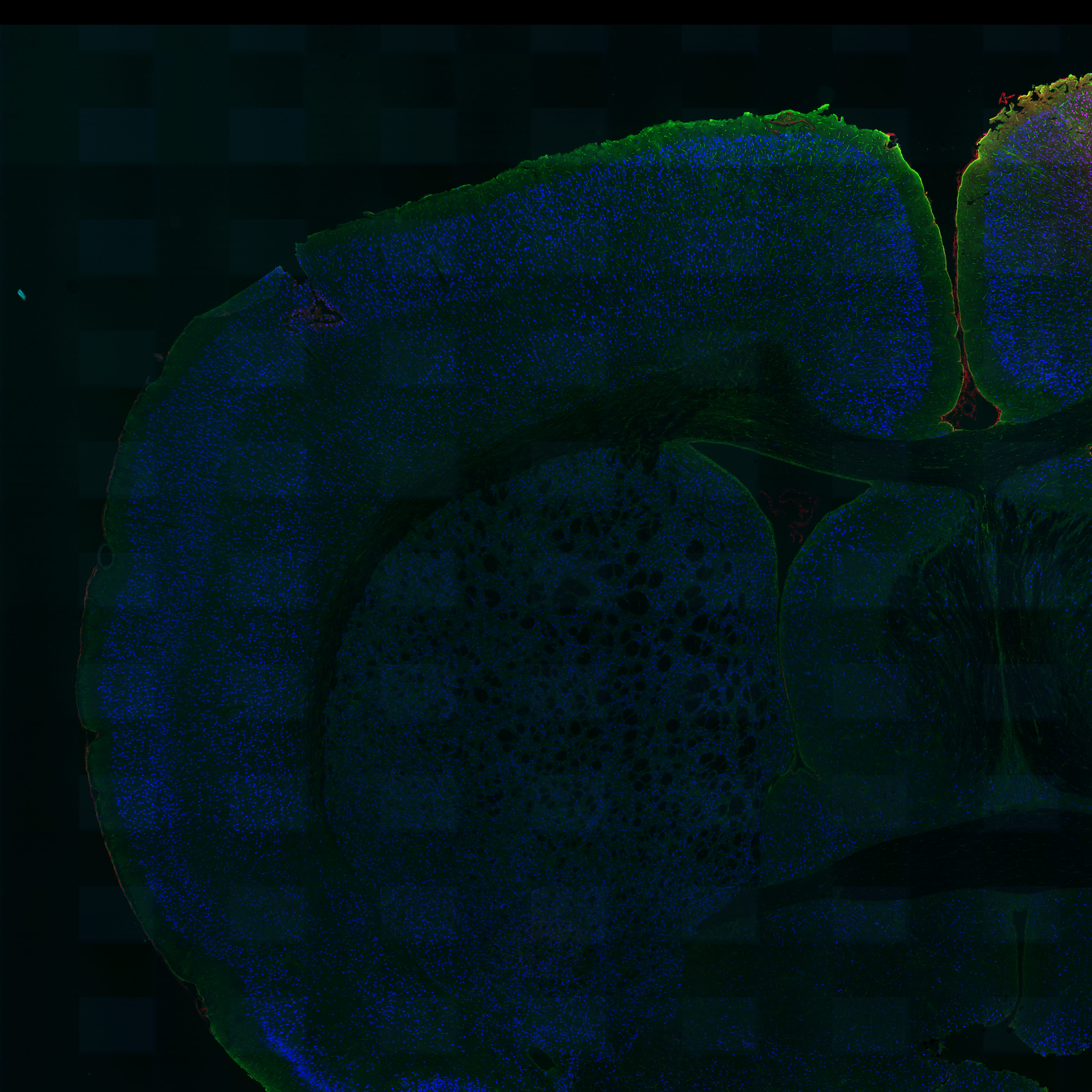

Supplement: Supplementary file 4 [file Presentation_1.ZIP › ET_Cover_slide~F-Spot000001.tiles/pano_11.jpg]

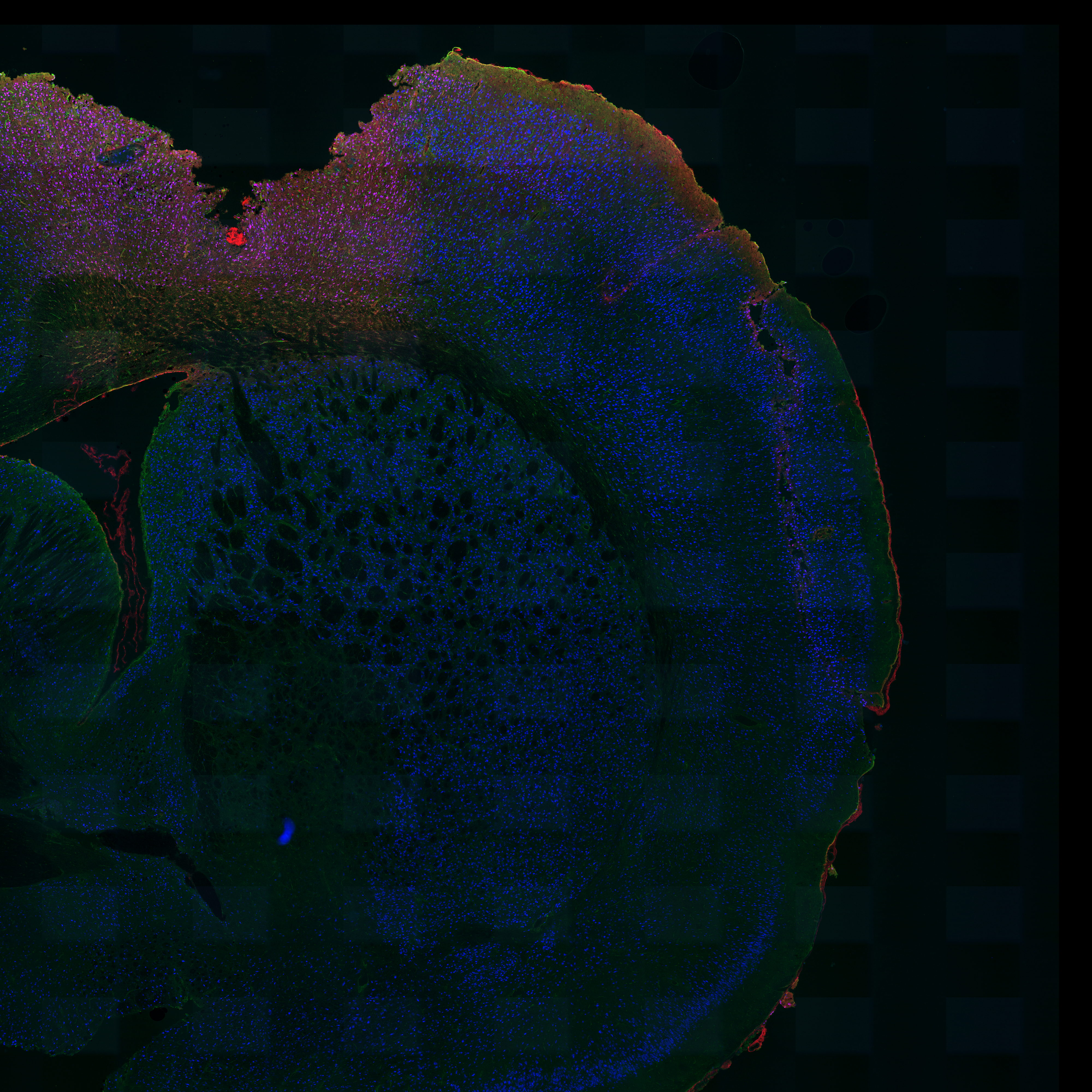

Supplement: Supplementary file 4 [file Presentation_1.ZIP › ET_Cover_slide~F-Spot000001.tiles/pano_12.jpg]

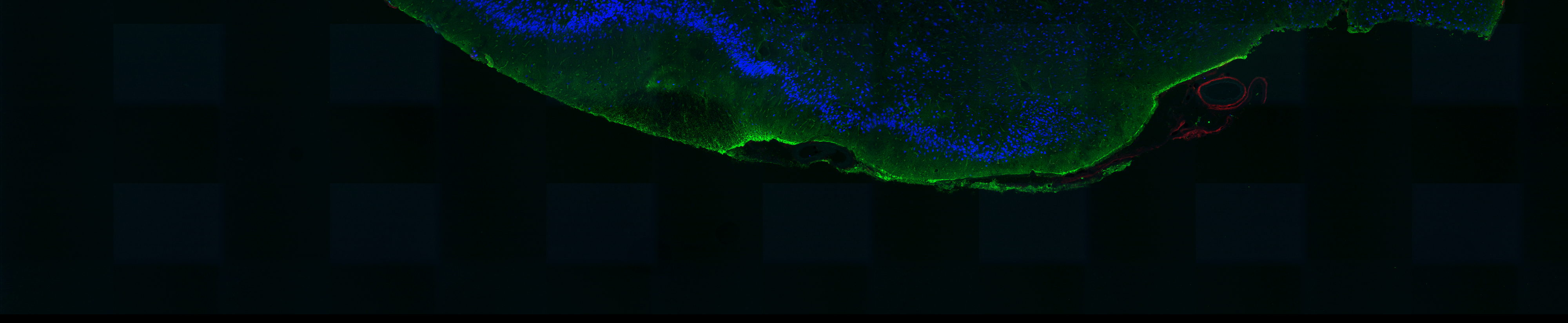

Supplement: Supplementary file 4 [file Presentation_1.ZIP › ET_Cover_slide~F-Spot000001.tiles/pano_21.jpg]

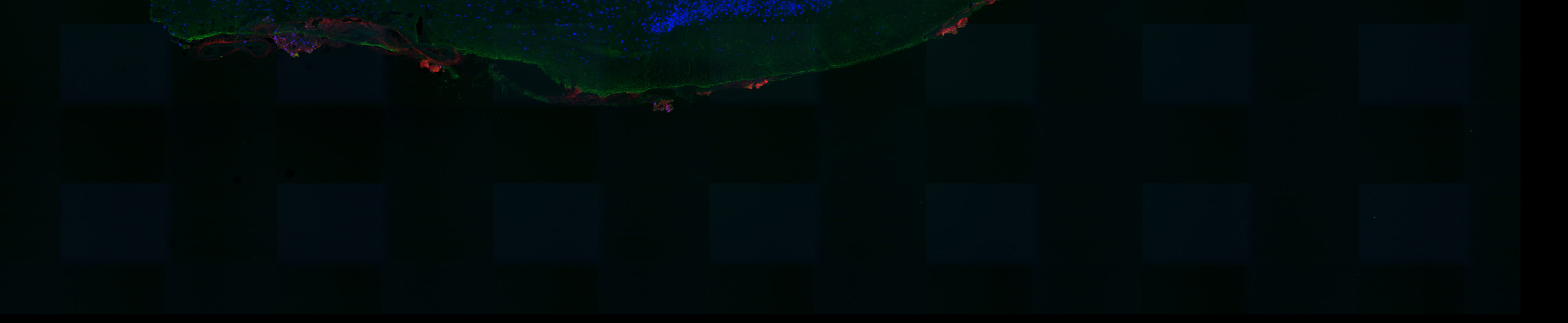

Supplement: Supplementary file 4 [file Presentation_1.ZIP › ET_Cover_slide~F-Spot000001.tiles/pano_22.jpg]

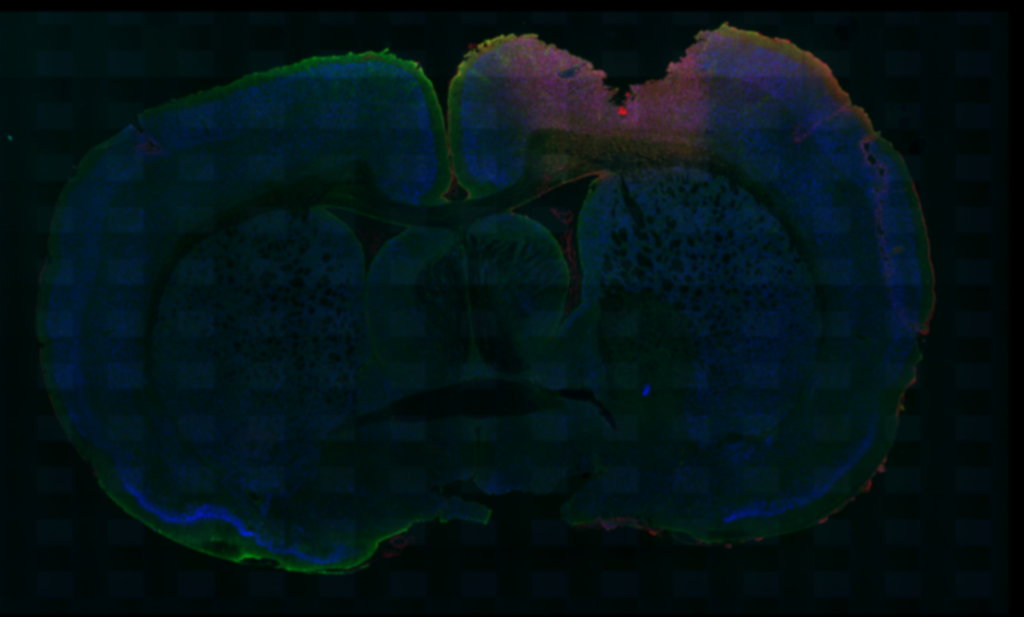

Supplement: Supplementary file 4 [file Presentation_1.ZIP › ET_Cover_slide~F-Spot000001.tiles/preview.jpg]

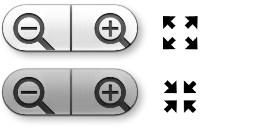

Supplement: Supplementary file 4 [file Presentation_1.ZIP › skin/androidbuttons.png]

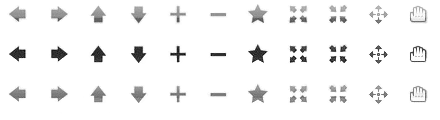

Supplement: Supplementary file 4 [file Presentation_1.ZIP › skin/buttons.png]

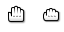

Supplement: Supplementary file 4 [file Presentation_1.ZIP › skin/drag-cursors.png]

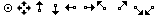

Supplement: Supplementary file 4 [file Presentation_1.ZIP › skin/qtvr-cursors.png]
