# Supplementary material for: Lesion Size Is Exacerbated in Hypoxic Rats Whereas Hypoxia-Inducible Factor-1 Alpha and Vascular Endothelial Growth Factor Increase in Injured Normoxic Rats: A Prospective Cohort Study of Secondary Hypoxia in Focal Traumatic Brain Injury
Source: Front Neurol. 2016 Mar 7;7:23. doi: 10.3389/fneur.2016.00023 (PMC4780037; doi:10.3389/fneur.2016.00023)
Supplement: Supplementary file 5 [file Data_Sheet_1.docx]

**Supplementary Material:**

# Lesion size is exacerbated in hypoxic rats whereas hypoxia-inducible factor 1 alpha and vascular endothelial growth factor increase in injured normoxic rats: a prospective cohort study of secondary hypoxia in focal traumatic brain injury.

*Eric Peter Thelin, Arvid Frostell, Jan Mulder, Nicholas Mitsios, Peter Damberg, Sahar Nikkhou Aski, Mårten Risling, Mikael Svensson, Maria Cristina Morganti-Kossmann, Bo-Michael Bellander.

*Correspondence: Eric Thelin

[Eric.thelin@ki.se](mailto:Eric.thelin@ki.se)

**Supplementary movie:**

This video demonstrates the surgical procedure including the intubation, pre-operative analgesia, the surgical procedure, per-operative monitoring, and post-operative procedures.

**Supplementary presentation/website:**

Link to an online section of a day 1 hypoxic brain with a lesion in the right parietal hemisphere (index.html). Please use the included interface to freely browse the section. Neurons are stained with NeuN (blue) and the complement protein C5b-9 (red). To stain vessels in the background, Aquaporin 4 was used (green) (1:200, AB3594, Millipore, Temecula, CA, USA). For other primary and secondary antibodies, please see Table 1. In order to open the presentation, please unzip all files to any folder and open “index.html”

**Supplementary Figure 1 - Anesthesiological set-up:**





Supplementary Figure 1. Gas mixtures (either 11% O_2_ or 22% O_2_) is transferred through a reservoir to the ventilator and then re-cycled to the animal. An O_2_-sensor is used to ensure that correct oxygen levels are provided. A pulsoxymeter device is used to monitor the animal.

# Supplementary Figure 2 – Omission of primary antibodies


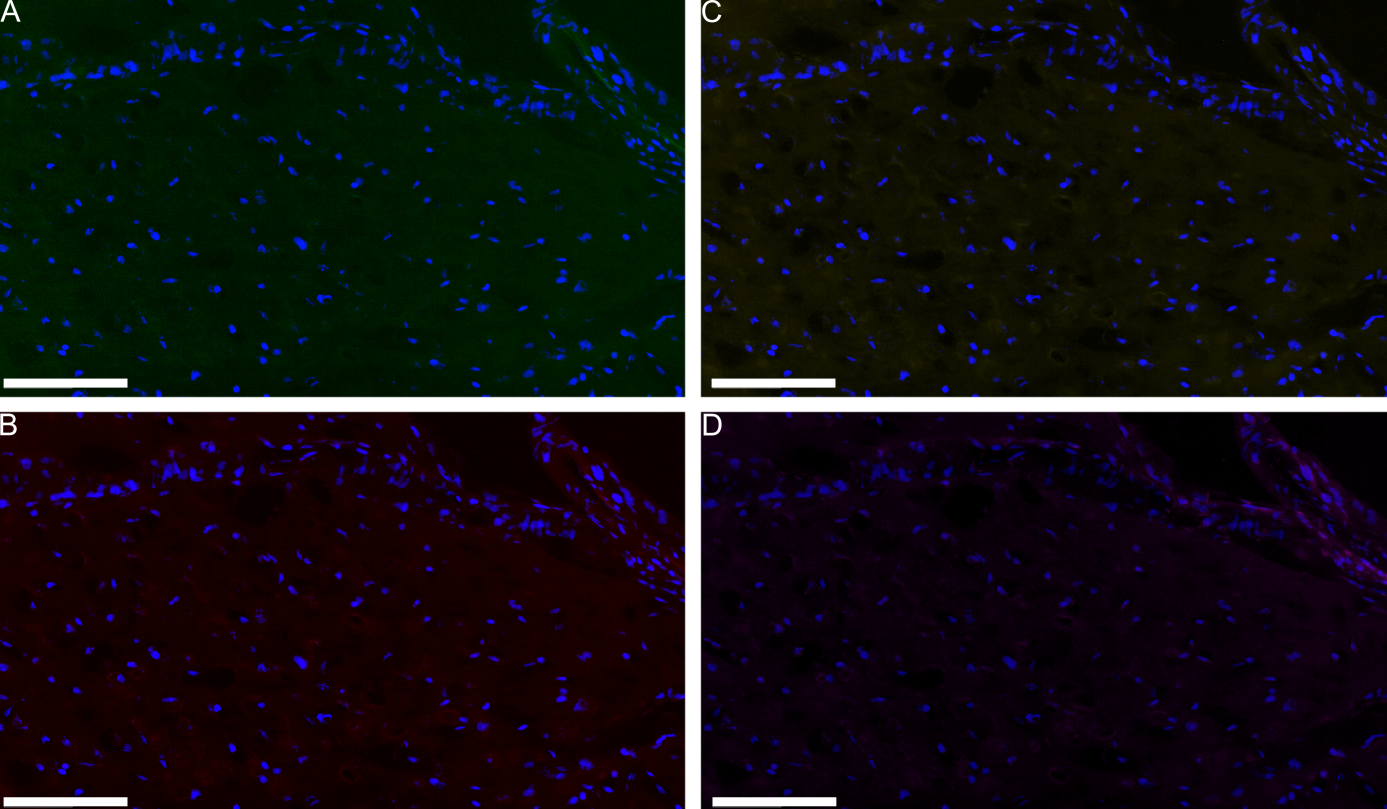


Supplementary Figure 2. Illustrating secondary antibodies omitting the primary antibodies from a trauma day 1 normoxic brain. **2A** Cy2 (anti-rabbit, 711-545-152, Jackson Laboratories, 1:200), **2B** Cy3 (anti-guinea pig, 706-165-148, Jackson Laboratories, 1:200), **2C** Cy3.5 (anti-goat, 705-585-147, Jackson Laboratories, 1:200), and **2D** Cy5 (anti-mouse, 715-175-150, Jackson Laboratories, 1:200). Exposure times were all set to 0.80, thus a lot higher than what was used in the experimental procedures and still no significant background was noted. DAPI (blue) was used as counterstain. Scale bar = 100µm.

# Supplementary Figure 3 – Correlation between lactate and pCO_2_


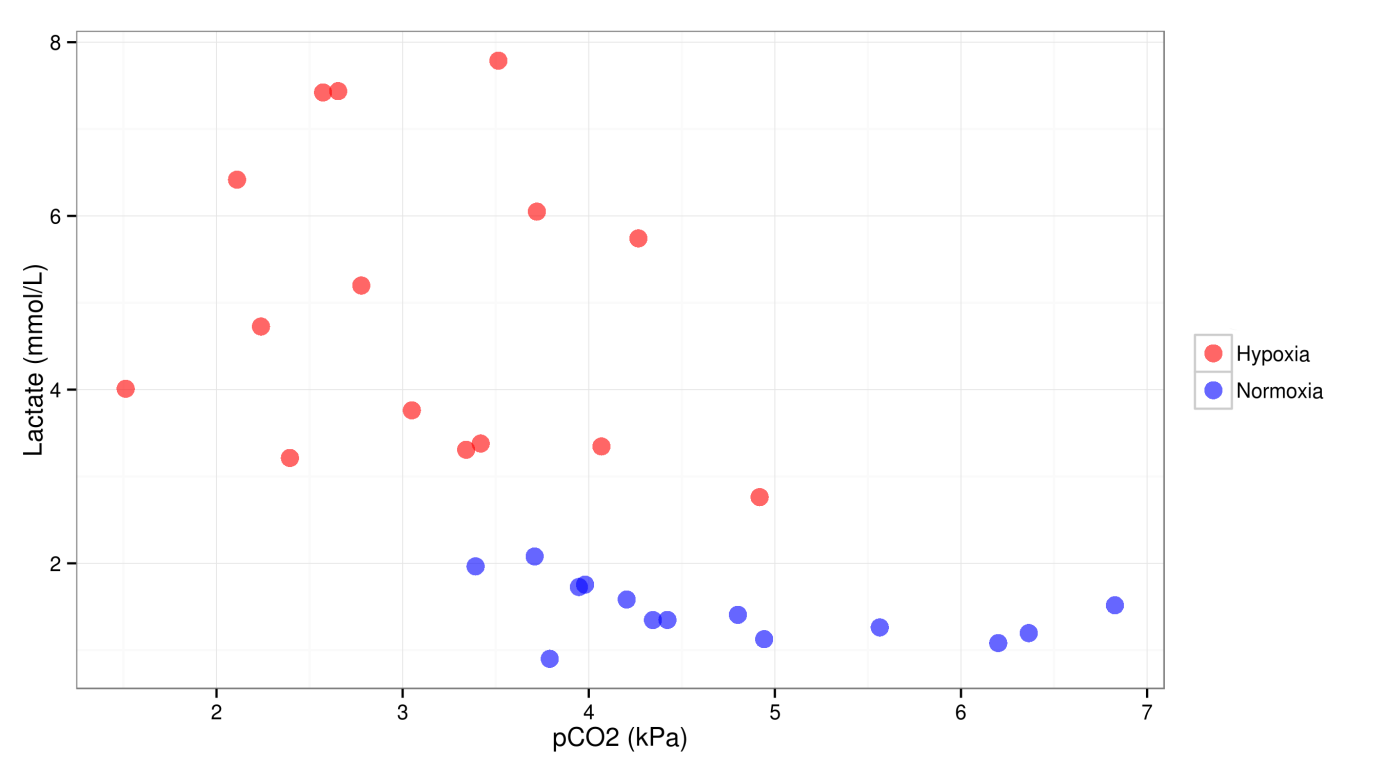


Supplementary Figure 3. Blood gas levels of lactate (y-axis) and pCO_2_ (x-axis). As the lactate levels increase, the pCO_2_ levels decrease, probably due to spontaneous hyperventilation because of metabolic acidosis in the hypoxic rats. This graph also shows that lactate levels in normoxic rats were never higher than in the lowest level detected in hypoxic rats.
